# The prognostic value of preoperative D-dimer to albumin ratio for overall survival and progression-free survival in colorectal cancer

**DOI:** 10.3389/fphys.2024.1369855

**Published:** 2024-02-29

**Authors:** Lishuang Wei, Hailun Xie, Qiwen Wang, Shuangyi Tang, Jialiang Gan

**Affiliations:** ^1^ Department of Colorectal and Anal Surgery, The First Affiliated Hospital, Guangxi Medical University, Nanning, Guangxi, China; ^2^ Guangxi Key Laboratory of Enhanced Recovery After Surgery for Gastrointestinal Cancer, Nanning, Guangxi, China; ^3^ Department of Pharmacy, The First Affiliated Hospital, Guangxi Medical University, Nanning, Guangxi, China

**Keywords:** D-dimer-to-albumin ratio, hypercoagulability, albumin, colorectal cancer, prognostic

## Abstract

**Introduction:** This study aimed to explore the predictive value of the D-dimer-to-albumin ratio (DAR) for progression-free survival (PFS) and overall survival (OS) in patients with colorectal cancer (CRC).

**Methods:** The Kaplan-Meier method was used to plot survival curves for PFS and OS. Receiver operating characteristic (ROC) curve analysis was used to evaluate the predictive efficacy of the DAR for PFS and OS in patients with CRC. Cox proportional hazards regression analysis was used to analyze prognostic factors influencing outcomes. A nomogram based on the DAR was constructed to predict 1-, 3-, and 5-year prognoses of patients with CRC; its predictive ability was evaluated using the concordance index (C-index) and calibration curves. Additionally, the clinical utility of the DAR-based nomogram was validated using an internal randomized validation cohort.

**Results:** A total of 1,339 patients with CRC who underwent surgery were enrolled. The optimal cut-off value for DAR was determined to be 3.320, dividing patients into low (<3.320 [n = 470]) and high (≥3.320 [n = 869]) DAR groups. Compared with other composite immune inflammatory markers, DAR exhibited superior prognostic predictive efficacy. Patients with a high DAR had a significantly worse prognosis than those with a low DAR (PFS, 50.9% *versus* [vs.] 69.4%, *p* < 0.001; OS, 52.9% vs. 73.8%, *p* < 0.001). DAR also demonstrated significant prognostic stratification for most tumor subgroups, particularly in the stage III-IV subgroup and normal carcinoembryonic antigen subgroup. DAR has been identified as an independent predictive indicator of PFS/OS in patients with CRC. For every standard deviation increase in DAR, the risk for PFS/OS in patients with CRC was reduced by 9.5% (hazard ratio [HR] 1.095 [95% confidence interval (CI) 1.013–1.185]; *p* = 0.022) and 9.3% (HR 1.093 [95% CI 1.012–1.180]; *p* = 0.024), respectively. The DAR-based nomogram was confirmed to demonstrate good prognostic prediction accuracy and achieved high evaluation in the internal validation cohort.

**Conclusion:** Preoperative DAR is a promising biomarker for predicting PFS and OS among patients with CRC. The DAR-based prognostic prediction nomogram may serve as an effective tool for the comprehensive assessment of prognosis in patients with CRC.

## Introduction

With the aging and growth of the global population, cancer has become one of the leading causes of death worldwide and poses a significant threat to life expectancy. Colorectal cancer (CRC) is one of the most common malignant tumors worldwide and a major contributor to cancer-related deaths (Sung et al., 2021; Siegel et al., 2022). The incidence of CRC in China is equally high. According to projections, CRC was expected to rank second among newly diagnosed cancers in China and fifth among cancer-related deaths by 2022 (Xia et al., 2022). Despite some progress in the diagnosis and treatment of CRC in recent years, most patients are diagnosed at advanced stages, and the mortality rate of advanced CRC remains high. Therefore, it is crucial to identify reliable tumor prognostic biomarkers to predict risk and make appropriate treatment decisions for patients with CRC.

Systemic inflammation plays an important role in the occurrence and progression of tumors because it is a part of the tumor microenvironment (Coussens and Werb, 2002; Candido and Hagemann, 2013; Diakos et al., 2014). Components of blood cells and proteins in the serum are simple and intuitive indicators of systemic inflammation in patients with tumors, including neutrophils, lymphocytes, albumin, and C-reactive protein (CRP), among others (Xie et al., 2022a; Xie et al., 2022b). Hypercoagulability is a common condition observed in patients with cancer and is believed to be associated with tumor angiogenesis, growth, and proliferation (Hu et al., 2014; Kawai and Watanabe, 2014; Lin et al., 2018). D-dimer, a marker of fibrinolysis and activation of the coagulation cascade, reflects hypercoagulable state. The correlation between CRC and D-dimer levels was first reported by Edwards et al. (1993). Studies have shown that elevated D-dimer levels are associated with metastasis, recurrence, and shorter survival in various cancers, including lung, stomach, ovarian, colorectal, and bladder cancers, among others (Li et al., 2020; Liu et al., 2020; Yamada et al., 2020; Kim and Song, 2021; Ma et al., 2021). Additionally, patients with cancer, especially those with gastrointestinal tumors, often experience severe malnutrition. Nutritional indicators are also important tools for assessing the prognosis of patients with tumors (Barreira, 2021). In clinical practice, serum albumin level is a simple and effective indicator that reflects patient nutritional status. Studies have shown that serum albumin levels are closely related to the prognosis of patients with gastrointestinal tumors (Antkowiak et al., 2019; Oh et al., 2019). Patients with low serum albumin levels generally have poor prognosis. Albumin is also considered to be an indicator of systemic inflammation. Under conditions of high inflammation, the synthesis of liver proteins changes rapidly, prioritizing the production of acute-phase proteins and leading to decreased albumin levels (Evans et al., 2021). Recently, Lin et al. combined D-dimer and albumin to construct the D-dimer-to-albumin ratio (DAR) and found that the preoperative DAR was an effective indicator for predicting the long-term prognosis of patients with gastric cancer (Lin et al., 2023). The DAR provides a comprehensive assessment of a patient’s inflammatory, nutritional, and tumor coagulation status and is considered to be a promising prognostic biomarker.

However, to our knowledge, there have been no studies addressing the relationship between DAR and the prognosis of patients with CRC. As such, the present retrospective cohort study aimed to investigate the prognostic value of DAR in predicting progression-free survival (PFS) and overall survival (OS) of patients with CRC. This study provides the first evidence that DAR, reflecting the coagulation status, is an effective biomarker for predicting recurrence and survival in CRC patients. These findings emphasize that coagulation function markers in serum serve as another important prognostic indicator in CRC patients and provide scientific evidence for the clinical application of DAR in CRC patients. Additionally, we constructed a predictive model based on DAR, which will be able to inform individualized and specific monitoring of the prognostic risk for patients with CRC, assisting physicians in developing more accurate treatment plans and management strategies for this patient population.

## Materials and methods

### Study population

Between 2015 and 2017, 1,339 patients with CRC, who underwent surgical treatment at the First Affiliated Hospital of Guangxi Medical University (Nanning China), were recruited. The inclusion criteria were as follows: histologically confirmed CRC; complete data regarding D-dimer and albumin levels, and other relevant factors; and age ≥18 years. DAR was constructed based on D-dimer and albumin, which can be influenced by thrombotic disorders and inflammation-related diseases, as well as neoadjuvant chemotherapy. Therefore, to minimize potential confounding factors, we excluded patients who underwent neoadjuvant chemotherapy before surgery, those with preexisting thrombotic disorders and/or receiving long-term anticoagulation therapy, autoimmune diseases, recent corticosteroid treatment, acute or chronic inflammatory diseases (such as acute upper respiratory tract infection, pneumonia, acute pancreatitis, acute appendicitis, or pyelonephritis). This study was approved and supported by the Ethics Committee of the First Affiliated Hospital of Guangxi Medical University.

### Data collection

Preoperative clinical data were collected through the electronic medical record system, including patient-, tumor-, and laboratory-related factors, and treatment information. In terms of patient-related factors, information, including sex, age, height, weight, hypertension, diabetes, and other relevant details was collected. These factors provide reference information regarding basic characteristics and overall health status. Regarding tumor-related factors, information regarding TNM staging (based on the eighth Edition of the American Joint Committee on Cancer [AJCC] tumor staging system), tumor location (colon and rectal), presence of perineural/vascular invasion, tumor size, and tumor differentiation was also collected. These factors serve as important indicators for assessing the severity, spread, and biological characteristics of tumors. In terms of treatment information, whether the patients underwent postoperative radiotherapy, chemotherapy, or other treatment modalities was recorded. These details can be analyzed in relation to the treatment efficacy and prognosis to evaluate the effectiveness of the treatment strategy. For laboratory-related factors, blood samples were obtained from patients within 7 days before surgery for testing. These laboratory-related factors include tumor markers, D-dimer, albumin, CEA, and other indicators. BMI was defined as weight (in kg) divided by the square of height (in meters); DAR was defined as the ratio of D-dimer (in µg/mL) to albumin (in g/L); The neutrophil-to-lymphocyte ratio (NLR) is defined as the ratio of neutrophils (in 10^9/L) to lymphocytes (in 10^9/L); The platelet-to-lymphocyte ratio (PLR) is defined as the ratio of platelets (in 10^9/L) to lymphocytes (in 10^9/L); The prognostic nutritional index (PNI) is defined as the serum albumin (in g/L) plus 5 times the lymphocytes (in 10^9/L); Normal CEA is defined as <5 ng/mL, while high CEA is defined as ≥5 ng/mL.

### Follow-up

Patients were followed up every 3 months for 2 years, then every 6 months for 3 years, and once per year thereafter. The final follow-up period was January 2023. Follow-ups were conducted through telephone consultations, and the patients attended regular outpatient visits for further examination. Study outcomes included OS and PFS. OS was calculated from the date of surgery to the date of last follow-up or death; PFS was defined as the time to local or distant disease recurrence. The median follow-up was 65.7 months (range, 1–106 months).

### Statistical analysis

Statistical analysis was performed using the R statistical software package, version 4.0.2 R Foundation for Statistical Computing, Vienna, Austria <http://cran.r-project.org/>. Continuous variables are expressed as mean ± standard deviation (SD) or median (interquartile range). Categorical variables are expressed as count (percentage). The chi-squared test or Fisher’s exact test was used to evaluate categorical variables, and the *t*-test was performed for continuous variables. To determine the optimal cut-off threshold for DAR, a receiver operating characteristic (ROC) curve was constructed using Harrell’s concordance index correction. Time-dependent ROC curve analysis was performed to compare the differences in the area under the ROC curve (AUC) for prognostic markers. OS and PFS were analyzed using the Kaplan–Meier (KM) method and log-rank tests (survival rates). A Cox proportional hazards regression model was employed to identify independent prognostic factors. The Schoenfeld residual test was conducted to examine the assumption of the Cox proportional hazards regression model and ensure its validity. R software was used to generate a nomogram based on variables that were significant in the Cox regression model. Internal validation curves, C-indices, and ROC curves were used to assess the discriminatory power and calibration of the predictive model. Decision curve analysis (DCA) was used to graphically compare the clinical benefits. Finally, differences with *p* < 0.05 were considered to be statistically significant.

## Results

### Demographic characteristics

The mean (±SD) age of the study population (840 male [62.7%], 466 female [36.6%]) was 58.28 ± 12.93 years. Among the cohort, 633 patients (47.3%) had colon cancer and 706 (52.7%) had rectal cancer. There were 368 (27.5%) cases of recurrence and 532 (39.7%) deaths. Clinicopathological staging revealed stages I-II in 713 (53.2%) and stages III-IV in 626 (46.8%) cases. DAR values ranged from 0.02 to 244.20 among all patients, with a mean of 8.37 ± 13.80 and a median of 4.58. Based on patient survival status, the AUC for DAR was 0.620, with a sensitivity of 43.0% and a specificity of 76.9%. The optimal cut-off value for DAR was determined to be 3.32 (Supplementary Figure S1). A DAR value of less than 3.32 is defined as low DAR, while a value of 3.32 or higher is considered high DAR.

Accordingly, patients were divided into low (<3.320 [n = 470]) and high (≥3.320 [n = 869]) DAR groups. A high DAR was significantly associated with female sex, advanced age, lower body mass index (BMI), higher frequency of colon cancer, larger tumor diameter, and higher carcinoembryonic antigen (CEA) levels. Compared with the low DAR group, the high DAR group had a later stage of cancer and a higher likelihood of metastasis. Furthermore, the overall mortality rate was 20.9% higher in the high DAR group than in the low DAR group (47.1% vs. 26.2%, respectively; *p* < 0.001). The high DAR group also exhibited an 8.6% higher recurrence rate than the low DAR group (21.9% vs. 30.5%, respectively; *p* = 0.001). Patients in the high DAR group had a 1-day longer hospital stay and incurred a cost 2,198.95 RMB higher than those in the low DAR group (Supplementary Table S1). The distribution of median DAR levels among various clinicopathological characteristics were explored. Female sex, age ≥60 years, low/normal BMI, recurrence, and death were associated with higher DAR in patients with CRC (Supplementary Figure S2).

### Comparison of composite immune inflammatory markers

To compare the predictive abilities of the DAR and other composite immune inflammatory markers for the prognosis of patients with CRC, ROC curves were plotted and AUCs were calculated. For 3-year PFS, the AUC for DAR was higher than that for NLR, PLR, and PNI (0.582 *versus* [vs.] 0.539 vs. 0.531 vs. 0.554, respectively). Similarly, for 5-year PFS, the AUC for DAR was also higher than that for NLR, PLR, and PNI (0.579 vs. 0.543 vs. 0.539 vs. 0.558). Compared with NLR (3-year OS, 0.564; 5-year OS, 0.549), PLR (3-year OS, 0.555; 5-year OS, 0.544), PNI (3-year OS, 0.567; 5-year OS, 0.560), and DAR (3-year OS, 0.607; 5-year OS, 0.594) demonstrated better prognostic predictive efficacy (Supplementary Figure S3).

### Survival differences in low-DAR *versus* high-DAR groups

**FIGURE 1:**
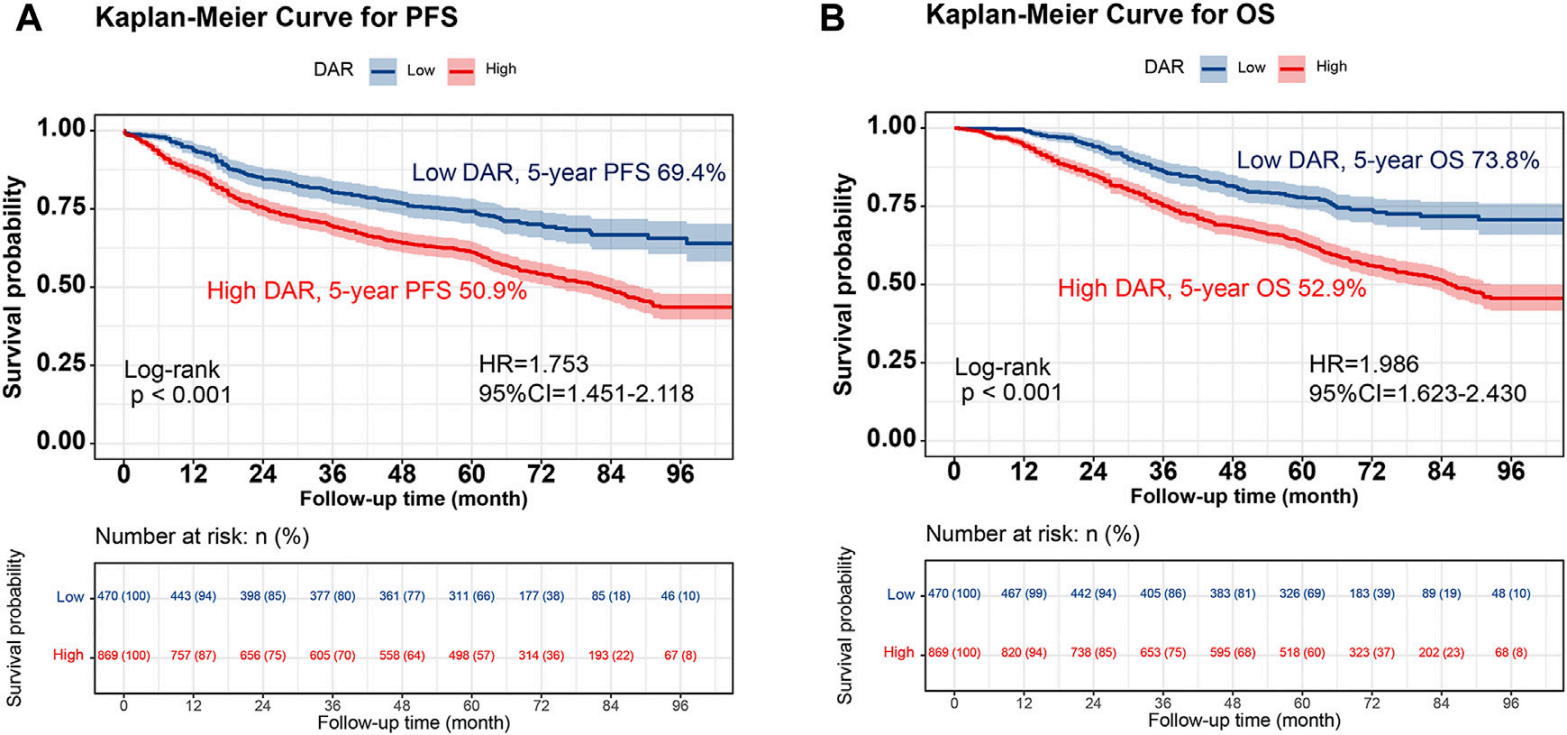
Kaplan-Meier curve of DAR in patients with colorectal cancer. Notes: **(A)**, PFS; **(B)**, OS. Abbreviation: DAR, D-Dimer to Albumin Ratio; PFS, Progression-free survival; OS, Overall survival; HR, Hazard ratio; CI, Confidence interval.

**FIGURE 2:**
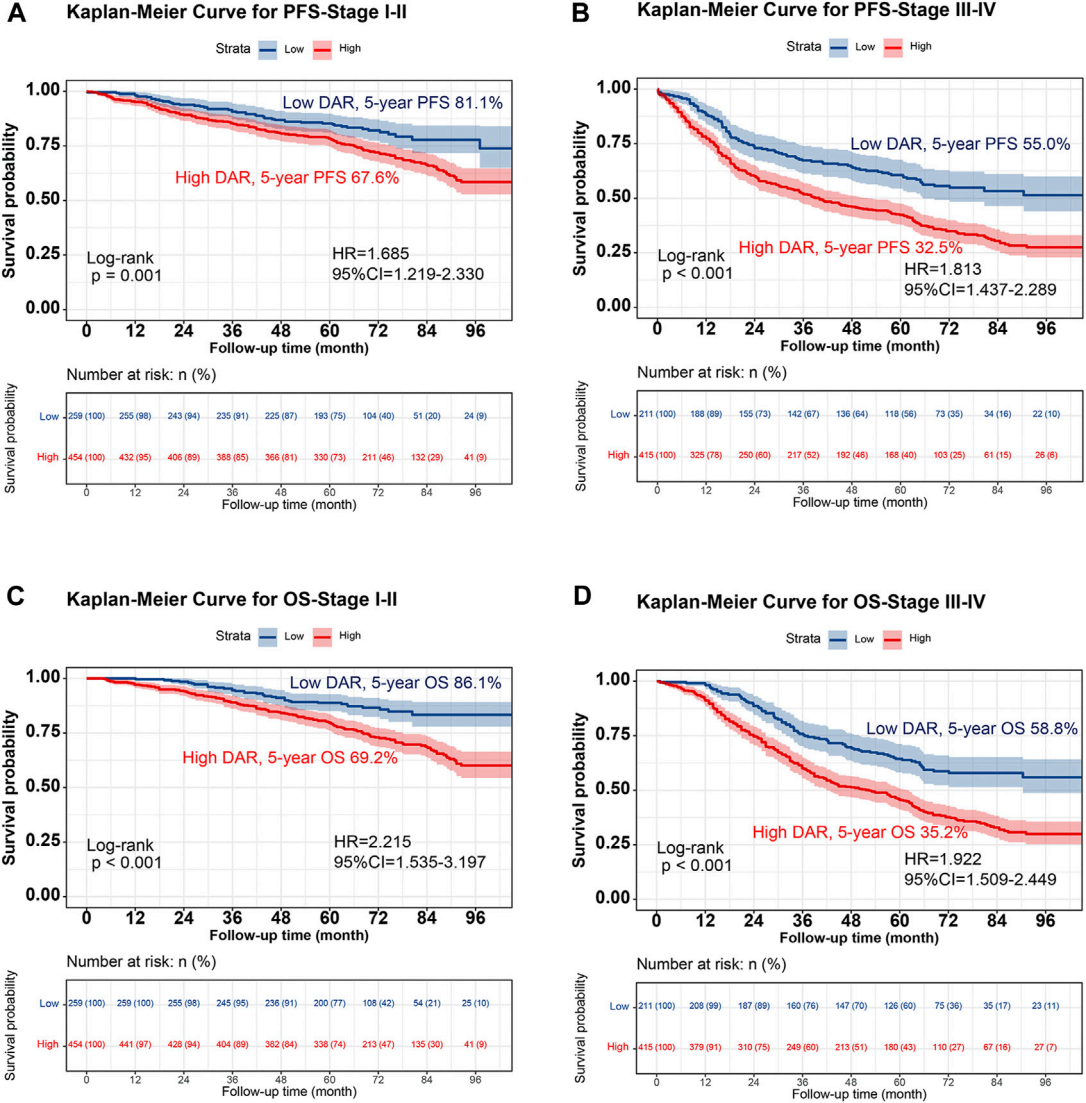
Stratified Kaplan-Meier curve of DAR based on TNM stage subgroup in patients with colorectal cancer. Notes: **(A)**, PFS (Stage I-II); **(B)**, PFS (Stage III-IV); **(C)**, OS (Stage I-II); **(D)**, OS (Stage III-IV). Abbreviation: DAR, D-Dimer to Albumin Ratio; PFS, Progression-free survival; OS, Overall survival; HR, Hazard ratio; CI, Confidence interval.

Kaplan-Meier analysis was performed to determine the correlation between DAR and the prognosis of patients with CRC. The results revealed that patients in the high DAR group had a worse prognosis, with a significantly lower 5-year survival rate than those in the low DAR group (PFS, 50.9% vs. 69.4% [*p* < 0.001]; OS, 52.9% vs. 73.8% [*p* < 0.001]) (Figure 1). Additionally, a subgroup analysis using Kaplan–Meier curves was performed. In the TNM staging subgroup, for both stages I-II and III-IV, the high-DAR group demonstrated poorer PFS than the low-DAR group (Figures 2A, B). Similar observations were made for OS (Figures 2C, D). For both colon and rectal cancers, the high-DAR group had shorter PFS and OS than the low-DAR group (Supplementary Figures S4, S5). The DAR effectively distinguished between PFS and OS in both the normal and high CEA subgroups, with superior differentiation observed in the normal CEA subgroup (Supplementary Figure S6).

### Prognostic values of DAR in patients with CRC

**FIGURE 3:**
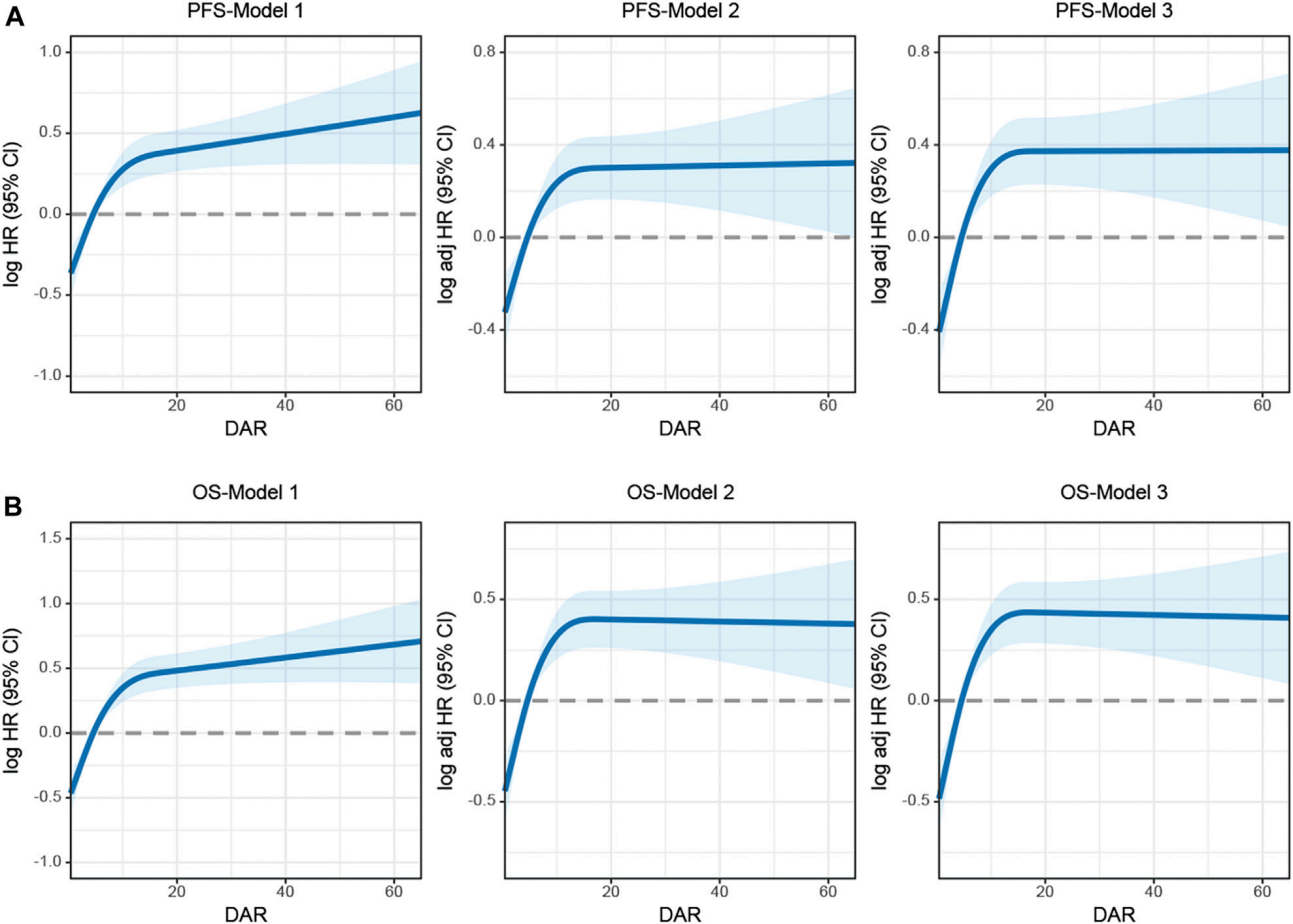
The association between DAR and survival in patients with colorectal cancer. Notes: **(A)**, PFS; **(B)**, OS. Model 1: No adjusted. Model 2: Adjusted for sex, age, and BMI. Model 3: Adjusted for sex, age, BMI, hypertension, diabetes, T stage, N stage, M stage, tumor size, location, perineural invasion, vascular invasion, differentiation, radiotherapy, chemotherapy. Abbreviation: DAR, D-Dimer to Albumin Ratio; PFS, Progression-free survival; OS, Overall survival.

Spline graphs were plotted with DAR on the *x*-axis and log hazard ratio (HR) (95% confidence interval [CI]) on the *y*-axis, which flexibly illustrated the association between DAR and PFS/OS in patients with CRC. A nonlinear relationship was found between DAR and PFS/OS in patients with CRC. As the DAR increased, the HR gradually increased, but stabilized after reaching approximately 10. This trend remained consistent across the different calibration models (Figures 3A, B). For every 1 SD increase in DAR, the risk for PFS in patients with CRC was reduced by 9.5% (HR 1.095 [95% CI 1.013–1.185]; *p* = 0.022). The high-risk group had a 78.7% higher risk for adverse PFS than the low-risk group (HR 1.787 [95% CI 1.454–2.196]; *p* < 0.001). A quartile analysis of DAR found that patients in the second, third, and fourth quartiles had adverse PFS rates that were 1.607, 1.578, and 1.982 times higher, respectively, than those in the first quartile (Table 1). Similarly, when exploring the relationship between DAR and OS using DAR as a continuous variable, every 1 SD increase in DAR resulted in a 9.3% decrease in adverse OS (HR 1.093 [95% CI 1.012–1.180]; *p* = 0.024). The high-risk group had a 92.9% higher risk for adverse OS than the low-risk group (HR 1.929 [95% CI 1.551–2.399]; *p* < 0.001). As the DAR increased, the HR for OS also gradually increased. The Q2 (2.59–4.58), Q3 (4.58–8.81), and Q4 (≥8.81) led to increased risk for adverse OS for patients (Table 2). The Schoenfeld residual analysis results indicated that in the Cox proportional hazards regression model, DAR did not exhibit statistical significance (*p* > 0.05), and the global test also lacked statistical significance. Therefore, we can conclude that these models meet the assumption of proportional hazards (Supplementary Figure S7). For PFS, a multivariable forest plot revealed that the DAR was an independent risk factor for the majority of patient subgroups (Supplementary Figure S8A). Similarly, patients with a high DAR had a relatively worse prognosis than those with a low DAR in most subgroups (Supplementary Figure S8B). Patients with CRC in the high DAR group had a higher recurrence rate than those in the low DAR group (30.5% vs. 21.9%) (Supplementary Table S1). Multivariable logistic regression analysis revealed that high DAR (≥3.320) was an independent risk factor affecting disease recurrence (OR 1.524 [95% CI 1.119–2.074]; *p* = 0.007) (Table 3).

**TABLE 1:** Association between DAR and PFS of patients with colorectal cancer.

DAR	Model a	*p*-value	Model b	*p*-value	Model c	*p*-value
Continuous (per SD)	1.166 (1.084,1.255)	<0.001	1.089 (1.007,1.177)	0.033	1.095 (1.013,1.185)	0.022
Cutoff value (High)	1.754 (1.452,2.119)	<0.001	1.654 (1.357,2.016)	<0.001	1.787 (1.454,2.196)	<0.001
Quartiles						
Q1 (∼2.59)	ref		ref		ref	
Q2 (2.59∼4.58)	1.554 (1.205,2.005)	0.001	1.55 (1.196,2.008)	0.001	1.607 (1.233,2.096)	<0.001
Q3 (4.58∼8.81)	1.481 (1.148,1.911)	0.002	1.476 (1.136,1.919)	0.004	1.578 (1.201,2.072)	0.001
Q4 (8.81∼)	2.045 (1.601,2.612)	<0.001	1.745 (1.341,2.271)	<0.001	1.982 (1.501,2.618)	<0.001
p for trend		<0.001		<0.001		<0.001

Notes: Model 1: No adjusted.

Model 2: Adjusted for sex, age, and BMI.

Model 3: Adjusted for sex, age, BMI, hypertension, diabetes, T stage, N stage, M stage, tumor size, location, perineural invasion, vascular invasion, differentiation, radiotherapy, chemotherapy.

**Abbreviation:** DAR, D-Dimer to Albumin Ratio; PFS, Progression-free survival; OS, overall survival.

**TABLE 2:** Association between DAR and OS of patients with colorectal cancer.

DAR	Model a	*p*-value	Model b	*p*-value	Model c	*p*-value
Continuous (per SD)	1.186 (1.102,1.277)	<0.001	1.095 (1.015,1.182)	0.019	1.093 (1.012,1.18)	0.024
Cutoff value (High)	1.987 (1.624,2.431)	<0.001	1.887 (1.529,2.328)	<0.001	1.929 (1.551,2.399)	<0.001
Quartiles						
Q1 (∼2.59)	ref		ref		ref	
Q2 (2.59∼4.58)	1.736 (1.321,2.283)	<0.001	1.777 (1.346,2.348)	<0.001	1.779 (1.339,2.364)	<0.001
Q3 (4.58∼8.81)	1.759 (1.341,2.308)	<0.001	1.811 (1.37,2.394)	<0.001	1.821 (1.365,2.43)	<0.001
Q4 (8.81∼)	2.455 (1.89,3.188)	<0.001	2.175 (1.645,2.875)	<0.001	2.297 (1.711,3.083)	<0.001
p for trend		<0.001		<0.001		<0.001

Notes: Model 1: No adjusted.

Model 2: Adjusted for sex, age, and BMI.

Model 3: Adjusted for sex, age, BMI, hypertension, diabetes, T stage, N stage, M stage, tumor size, location, perineural invasion, vascular invasion, differentiation, radiotherapy, chemotherapy.

**Abbreviation:** DAR, D-Dimer to Albumin Ratio; PFS, Progression-free survival; OS, overall survival.

**TABLE 3:** Association between DAR and recurrence of patients with colorectal cancer.

DAR	Model a	*p*-value	Model b	*p*-value	Model c	*p*-value
Continuous (per SD)	1.032 (0.899,1.185)	0.651	0.91 (0.77,1.076)	0.271	0.93 (0.78,1.11)	0.415
Cutoff value (High)	1.563 (1.203,2.031)	<0.001	1.390 (1.036,1.865)	0.028	1.524 (1.119,2.074)	0.007
Quartiles						
Q1 (∼2.59)	ref		ref		ref	
Q2 (2.59∼4.58)	1.593 (1.125,2.257)	0.009	1.538 (1.051,2.250)	0.027	1.568 (1.063,2.314)	0.023
Q3 (4.58∼8.81)	1.324 (0.929,1.887)	0.121	1.255 (0.849,1.855)	0.254	1.329 (0.886,1.994)	0.169
Q4 (8.81∼)	1.571 (1.109,2.226)	0.011	1.177 (0.786,1.764)	0.429	1.359 (0.886,2.083)	0.160
p for trend		0.030				

Notes: Model 1: No adjusted.

Model 2: Adjusted for sex, age, and BMI.

Model 3: Adjusted for sex, age, BMI, hypertension, diabetes, T stage, N stage, M stage, tumor size, Location, perineural invasion, vascular invasion, differentiation, radiotherapy, chemotherapy.

**Abbreviation:** DAR, D-Dimer to Albumin Ratio; PFS, Progression-free survival; OS, overall survival.

### Establishment of DAR-based prediction nomograms

Using multivariate Cox regression analysis, 7 independent prognostic factors that influenced PFS and OS were identified using regression analysis. These factors included age, T stage, N stage, M stage, vascular invasion, CEA, and DAR (Supplementary Tables S2, S3). Based on these key determining factors, DAR-based nomograms were constructed to predict the PFS and OS in patients with CRC. The predicted probabilities of PFS and OS at 1, 3, and 5 years were calculated by summing the scores for each variable. Higher total scores were associated with lower PFS and OS probabilities (Supplementary Figures S9, S10). The 1-, 3-, and 5-year AUC for the PFS/OS nomograms were (0.807, 0.776, and 0.764) and (0.760, 0.781, and 0.769), respectively (Supplementary Figures S12A, B). The C-index of the nomograms were 0.721 and 0.730, respectively. According to the calibration curves, there was good consistency between the actual and predicted probabilities of 1-, 3-, and 5-year PFS (Supplementary Figure S12A) and OS (Supplementary Figure S12B). DCA was performed to compare the clinical benefits of DAR-based nomograms and traditional tumor staging. The results showed that DAR-based nomograms provided better clinical benefits than traditional tumor staging for both PFS and OS in the 1–5 year period (Supplementary Figures S13A, B). Furthermore, patients were categorized into high- and low-scoring groups based on the median scores from the nomogram. The results demonstrated that the high-score group had significantly worse PFS/OS compared to the low-score group (Supplementary Figures S14A, B).

### Validation of the DAR-based prediction models

**FIGURE 4:**
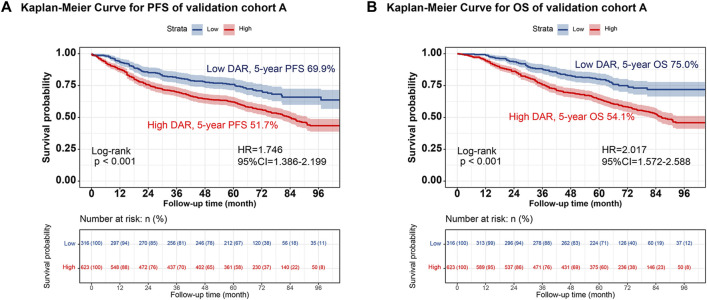
The validation cohort A of Kaplan-Meier curve of DAR. Notes: **(A)**, PFS; **(B)**, OS. Abbreviation: DAR, D-Dimer to Albumin Ratio; PFS, Progression-free survival; OS, Overall survival; HR, Hazard ratio; CI, Confidence interval.

**FIGURE 5:**
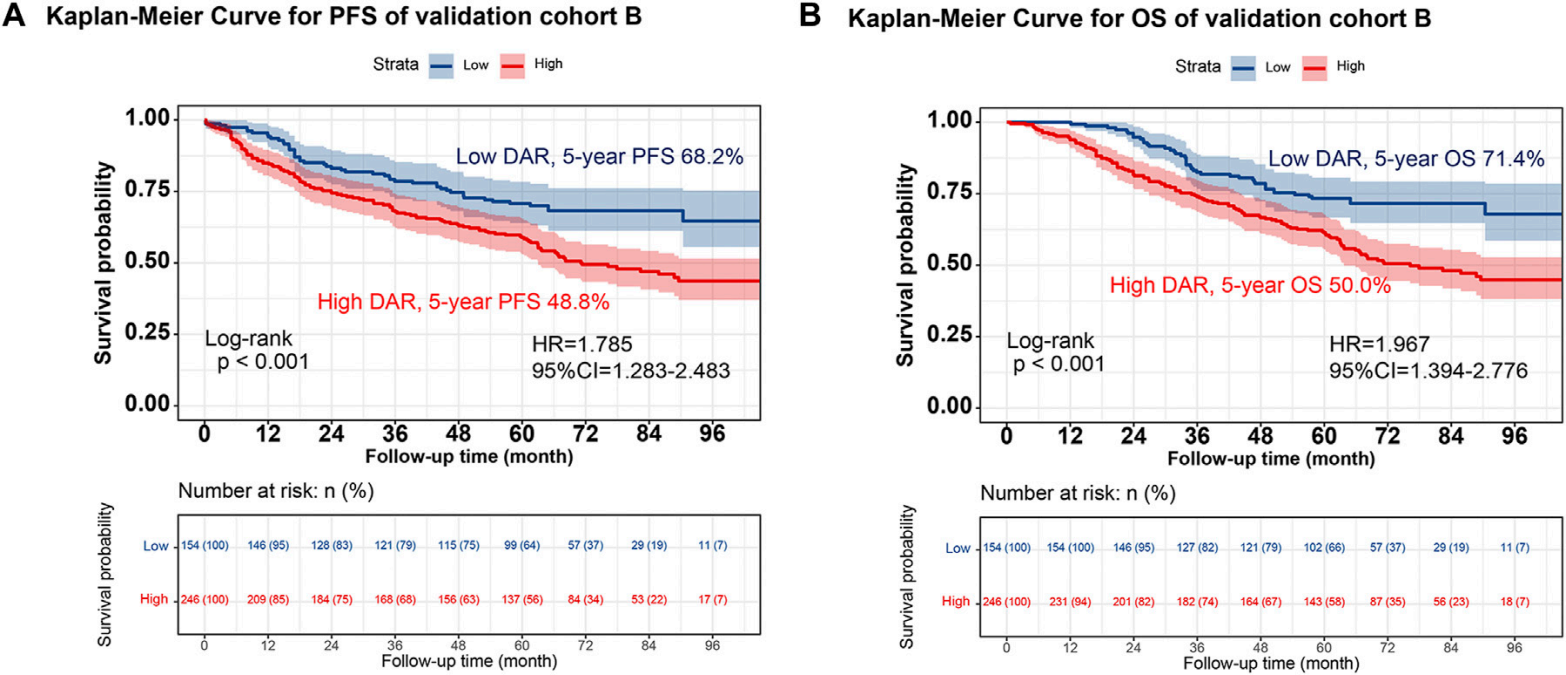
The validation cohort B of Kaplan-Meier curve of DAR. Notes: **(A)**, PFS; **(B)**, OS. Abbreviation: DAR, D-Dimer to Albumin Ratio; PFS, Progression-free survival; OS, Overall survival; HR, Hazard ratio; CI, Confidence interval.

Individuals were randomly selected for internal validation in a 7:3 ratio and divided into validation cohorts A (n = 939) and B (n = 400) (Supplementary Table S4). No significant differences were observed between the 2 groups. Overall, the results observed in the validation cohort were similar to the overall results. In validation cohort A, DAR effectively stratified the PFS/OS of patients with CRC (Figure 4). In validation cohort B, patients with CRC in the high-DAR group had a lower 5-year survival rate than those in the low-DAR group (PFS, 48.8% vs. 68.2% [*p* < 0.001]; OS, 50.0% vs. 71.4% [*p* < 0.001]) (Figure 5). The C-indices for PFS/OS in validation cohort A were 0.726 and 0.736, respectively. In validation cohort B, the C-indices for PFS/OS was 0.719 and 0.723, respectively. The ROC curves for both validation cohorts A and B demonstrated good accuracy, with all AUCs >0.75 (Supplementary Figure S15). The calibration curves for the validation cohort demonstrated good consistency between the actual and predicted probabilities of 1-, 3-, and 5-year PFS (Supplementary Figures S16A, C) and OS (Supplementary Figures S16B, D). In validation cohort A, DCA revealed that the clinical benefits of DAR-based nomograms were superior to those of the traditional tumor stage for 1-, 3-, and 5-year PFS/OS (Supplementary Figures S17A, B). The same phenomenon was observed in validation cohort B (Supplementary Figures S17C, D). Kaplan–Meier curves for the nomograms in the validation cohorts demonstrated that individuals with a high score exhibited lower PFS/OS than those with a low score (Supplementary Figure S18).

## Discussion

D-dimer is a degradation product of fibrinogen, which is activated and degraded when clots form in blood vessels, releasing D-dimer (Weitz et al., 2017). Venous thromboembolism (VTE) is one of the leading causes of morbidity and mortality in patients with cancer because cancer cells have direct and indirect effects on the microenvironment that promote blood clot formation, clinically manifested as thrombosis (Donnellan and Khorana, 2017). A study by Heit et al. (Heit et al., 2000) reported that systemic chemotherapy can increase the risk for VTE in patients by a factor of 2–6. Anticoagulant therapy for the prevention of VTE and cancer-associated thrombosis has become an important component of management of patients with cancer. A prospective study proposed that biomarkers, such as D-dimer and high-sensitivity CRP, may play a role in determining the optimal duration of anticoagulant therapy for cancer patients with thrombosis (Jara-Palomares et al., 2018). Albumin is one of the most abundant blood proteins and plays a crucial role in maintaining homeostasis and facilitating the transport of nutrients and drugs. Tumors can induce inflammation and metabolic changes, leading to an imbalance in protein synthesis and degradation. In some cases, this can result in hypoalbuminemia and decreased blood albumin levels in the blood (Don and Kaysen, 2004; Nazha et al., 2015; Evans et al., 2021). Malignant tumors consume significant amounts of nutrients, including proteins. In this scenario, tumor cells take up and utilize proteins, which can lead to a reduction in albumin levels in the body. By combining the advantages of D-dimer and albumin, DAR is a promising indicator for predicting tumor prognosis.

An elevated DAR may reflect changes in the coagulation state caused by tumor-induced inflammation and vascular endothelial injury. Tumor cells can produce inflammatory cytokines and procoagulant factors, leading to an increase in D-dimer levels and potentially affecting plasma albumin levels. However, the relationship between DAR and tumors remains under investigation, and there is currently insufficient evidence to determine a direct correlation between them. Some studies have suggested that DAR may serve as an adjunct prognostic indicator or assessment tool for tumor progression in specific types of tumors. Zhang et al. found that the albumin-to-D-dimer ratio is a useful indicator of chemotherapy efficacy and prognosis in patients with advanced gastric cancer undergoing first-line chemotherapy. Low albumin and high D-dimer levels are associated with poor prognosis (Zhang et al., 2021). Lin et al. demonstrated that the preoperative DAR based on the plasma D-dimer index and albumin level was a promising biomarker for predicting the long-term prognosis of patients with gastric cancer (Lin et al., 2023). However, there is currently no research exploring the relationship between DAR and the prognosis of patients with CRC.

In this study, we found that DAR was an independent predictive indicator of PFS/OS in patients with CRC. The high DAR group had a significantly higher risk for poor PFS and OS than the low DAR group, with an increase of 78.7% and 92.9%, respectively. The DAR can also serve as an adjunct tool for pathological staging, enabling effective prognostic differentiation among patients with the same pathological stage. We observed that a high DAR was associated with adverse patient characteristics (advanced age and lower BMI) and more aggressive tumor features (larger tumor diameter, higher CEA level, and later pathological stage). Furthermore, the high DAR group had a higher likelihood of recurrence. Multivariate logistic regression analysis revealed that high DAR was an independent risk factor for recurrence in patients with CRC. Collectively, our results suggest that DAR is a valuable indicator for predicting the prognosis of patients with CRC and can provide guidance for personalized treatment in clinical settings.

It is important to note that a single indicator cannot be used to comprehensively evaluate changes in the condition and prognosis of patients with cancer. Therefore, prognostic assessment of patients with cancer requires a comprehensive consideration of clinical symptoms, signs, imaging examinations, histopathology, and other aspects. To address these issues, we used Cox regression to identify independent prognostic variables for patients with CRC and constructed a DAR-based nomograms. We confirmed the good predictive accuracy of the DAR-based nomograms using the C-index, ROC curve, and calibration curve. Compared with traditional TNM staging, the DAR-based nomograms yielded better clinical benefits. Furthermore, we validated the effectiveness of the DAR-based nomograms in predicting the prognosis of patients with CRC using an internal validation cohort.

This study employed a retrospective, single-center design, which may have certain limitations due to the constraints of sample source. Single-center studies typically include patients from a single institution, which can introduce geographical bias and limit the external validity of the results. Additionally, retrospective studies often rely on historical data and records, posing risks of incomplete or inaccurate information retrieval, thus impacting the reliability and generalizability of the findings. Overall, the generalizability of the results to a broader population may face challenges in single-center and retrospective studies. Therefore, future prospective, multicenter studies are still needed to validate the findings of this research. Moreover, our sample size was limited, and multicenter studies could provide larger sample sizes for further research. Lastly, although internal validation was performed in this study, extensive external validation is still necessary prior to the clinical application of DAR-based nomograms.

## Conclusion

Preoperative DAR, based on plasma D-dimer and albumin levels, is a promising biomarker for predicting PFS and OS of patients with CRC. The DAR-based prognostic prediction nomogram may serve as an effective tool for the comprehensive assessment of prognosis in patients with CRC.

## Data Availability

The original contributions presented in the study are included in the article/Supplementary Material, further inquiries can be directed to the corresponding authors.
