# Supplementary material for: The prognostic value of preoperative D-dimer to albumin ratio for overall survival and progression-free survival in colorectal cancer
Source: Front Physiol. 2024 Feb 29;15:1369855. doi: 10.3389/fphys.2024.1369855 (PMC10937459; doi:10.3389/fphys.2024.1369855)
Supplement: Supplementary file 1 [file DataSheet1.docx]

**Figure S1.** The optimal threshold cutoff of DAR by receiver operator characteristic curve.

**
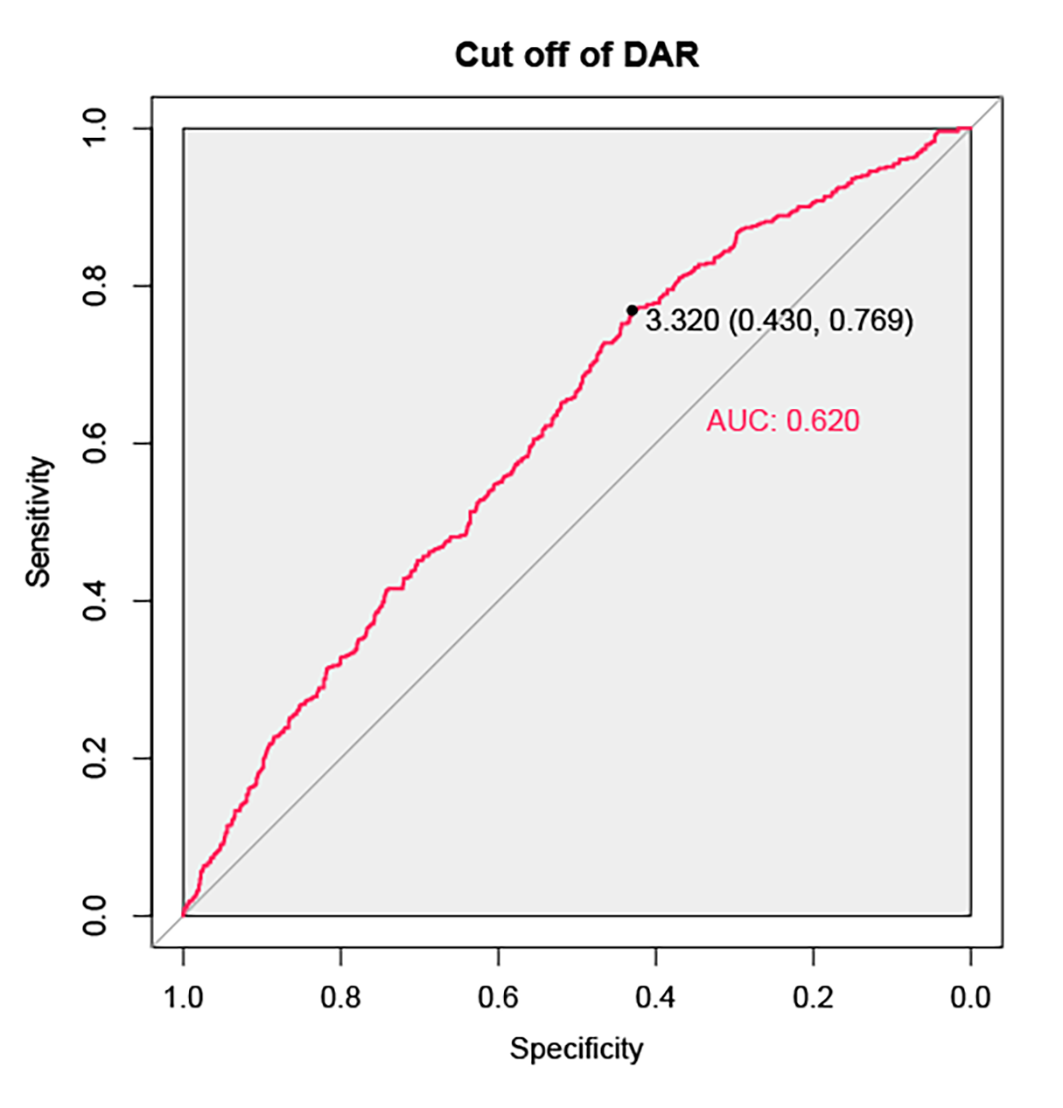
**

**Abbreviation:** DAR, D-Dimer to Albumin Ratio.

**Figure S2.** Median DAR of clinicopathological characteristics.

**
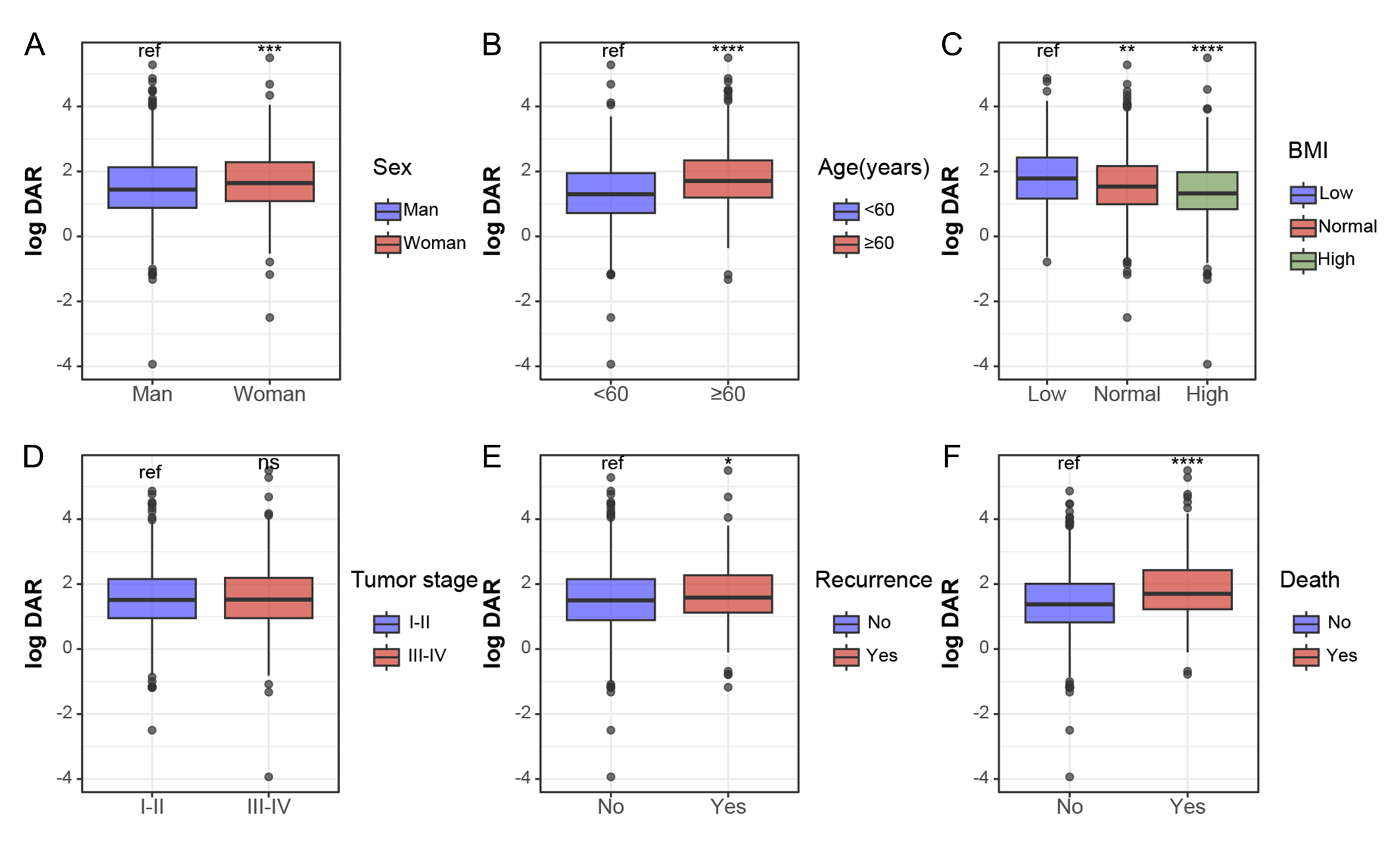
**

**Notes:** A, sex; B, age; C, BMI; D, tumor stage; E, recurrence; F, death.

**Abbreviation:** DAR, D-Dimer to Albumin Ratio; BMI, Body mass index.

**Figure S3.** Comparison of the prognostic value of composite immune inflammatory markers by receiver operator characteristic curve.

**
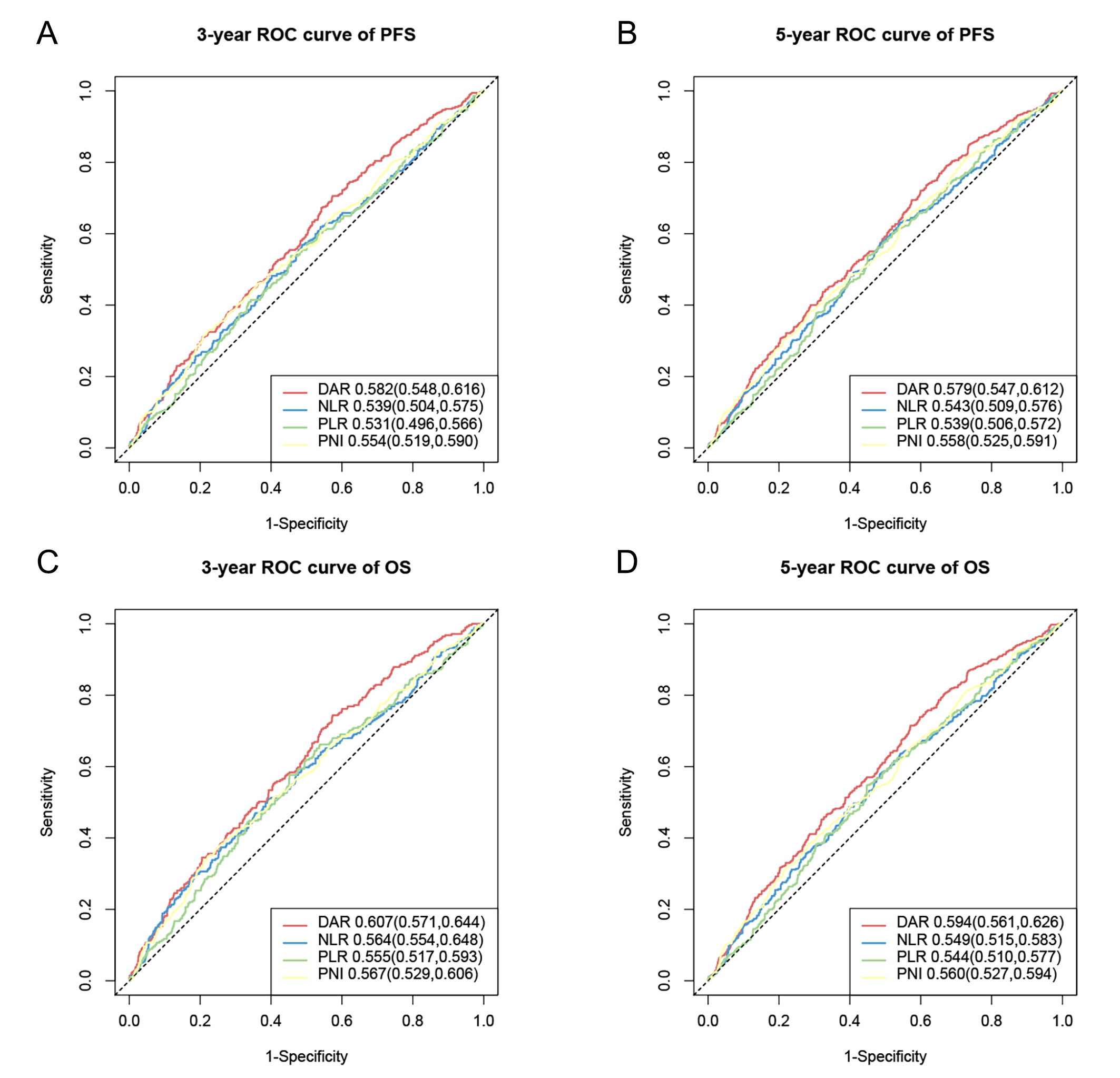
**

**Notes:** A, 3-year of PFS; B, 5-year of PFS; C, 3-year of OS; C, 5-year of OS.

**Abbreviation:** DAR, D-Dimer to Albumin Ratio; NLR, Neutrophil-to-lymphocyte ratio; PLR, Platelet-to-lymphocyte ratio; PNI, prognostic nutritional index; PFS, Progression-free survival; OS, Overall survival; ROC, Receiver operator characteristic curve.

**Figure S4.** Stratified Kaplan-Meier curve of DAR of patients with colon cancer.


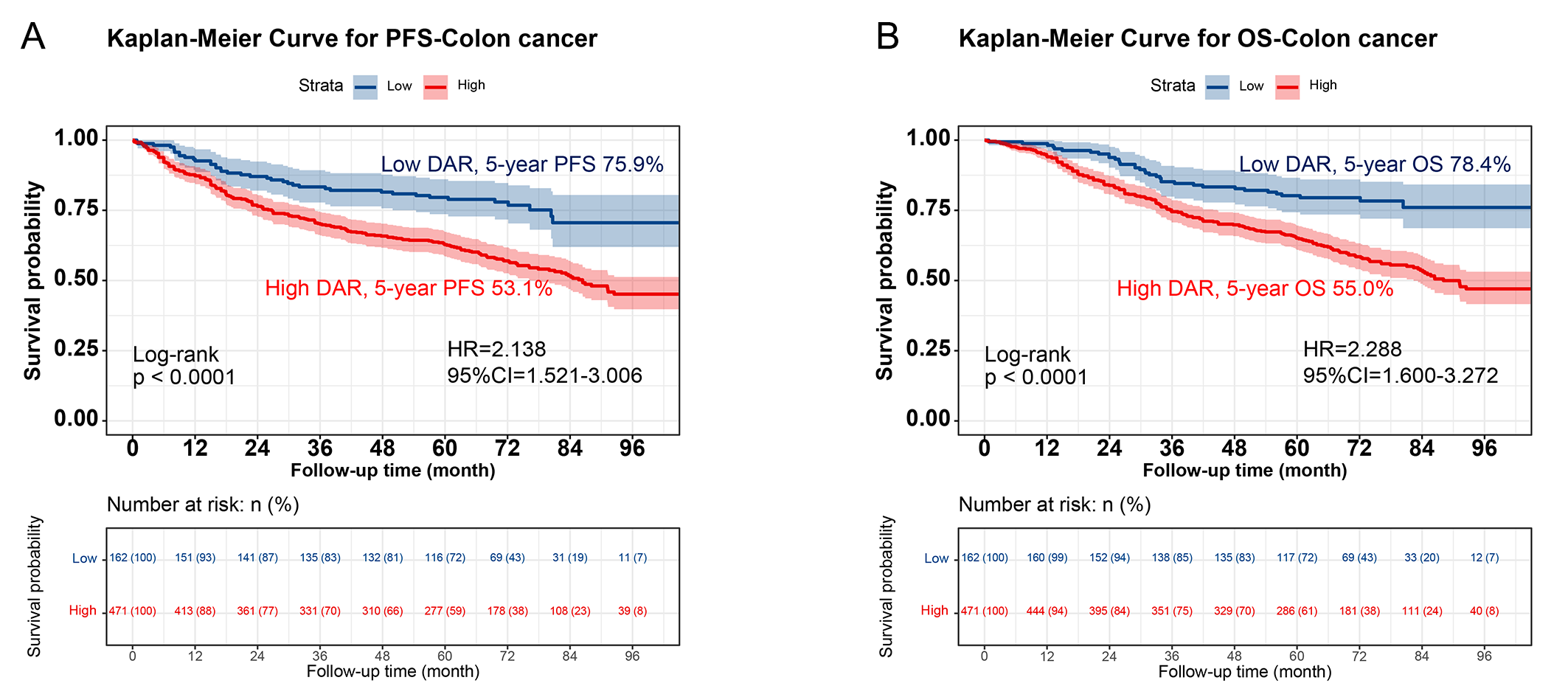


**Notes:** A, PFS; B, OS.

**Abbreviation:** DAR, D-Dimer to Albumin Ratio; PFS, Progression-free survival; OS, Overall survival; HR, Hazard ratio; CI, Confidence interval.

**Figure S5.** Stratified Kaplan-Meier curve of DAR of patients with rectal cancer.


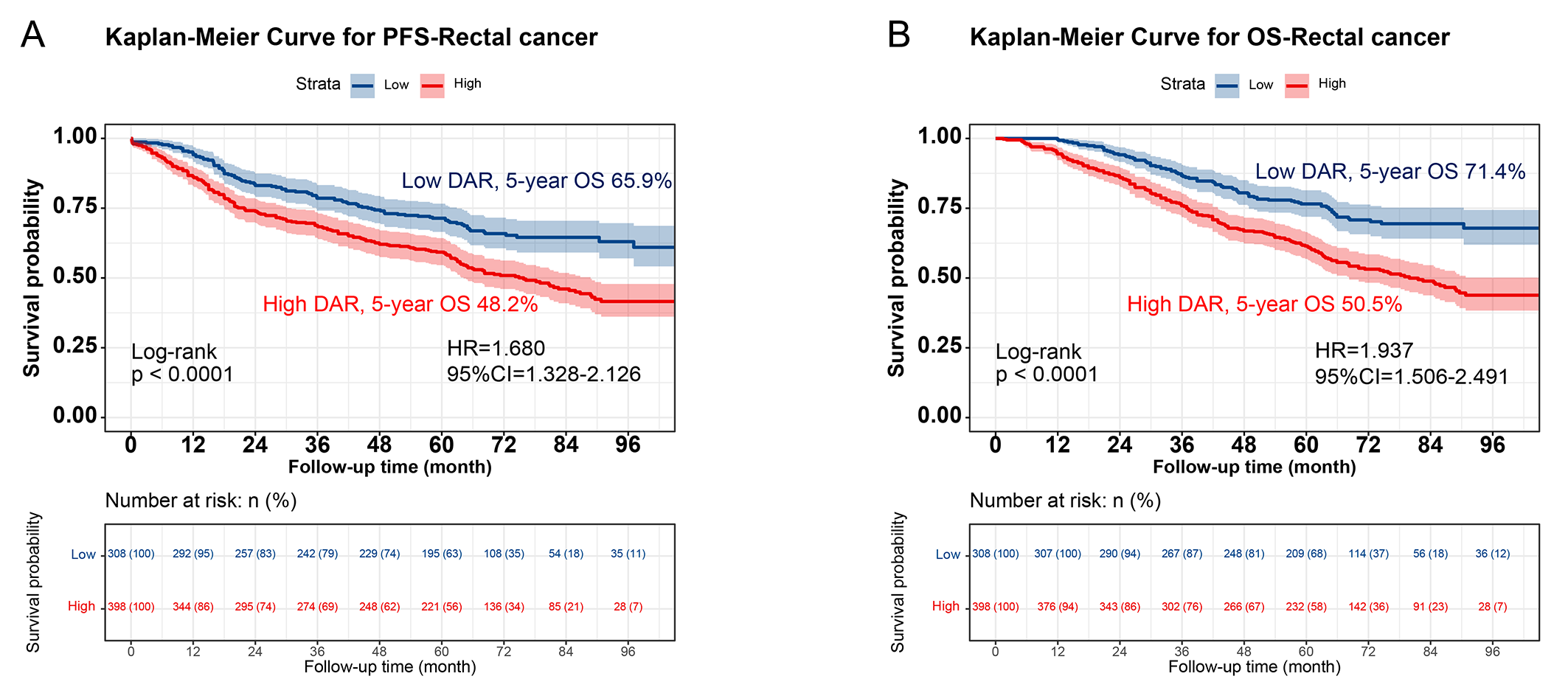


**Notes:** A, PFS; C, OS.

**Abbreviation:** DAR, D-Dimer to Albumin Ratio; PFS, Progression-free survival; OS, Overall survival; HR, Hazard ratio; CI, Confidence interval.

**Figure S6.** Stratified Kaplan-Meier curve of DAR based on CEA subgroup.


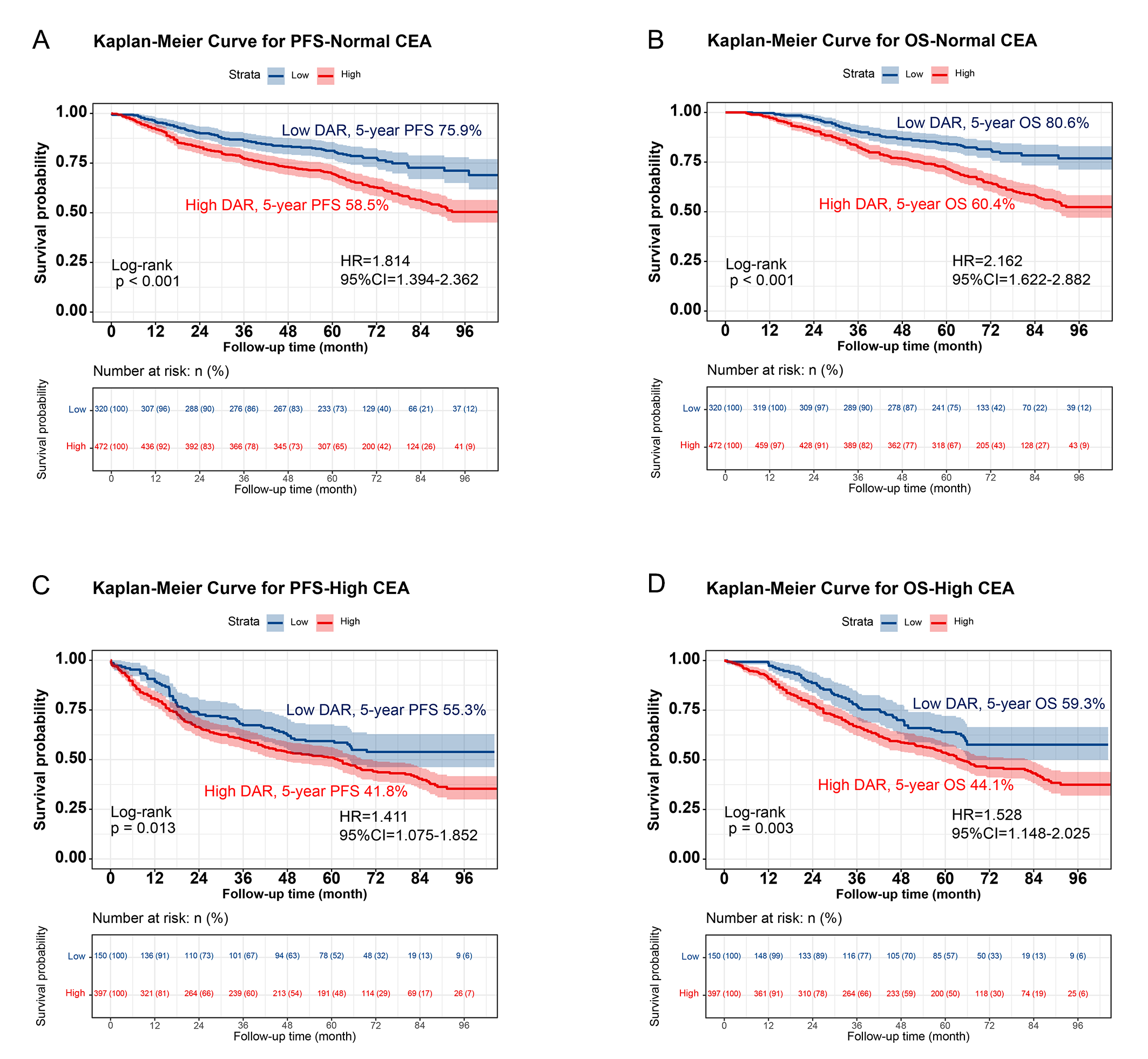


**Notes:** A, PFS-normal CEA; B, OS-normal CEA; C, PFS-high CEA; C, OS-high CEA.

**Abbreviation:** DAR, D-Dimer to Albumin Ratio; PFS, Progression-free survival; OS, Overall survival; HR, Hazard ratio; CI, Confidence interval.

**Figure S7.** The Schoenfeld residual analysis.

**
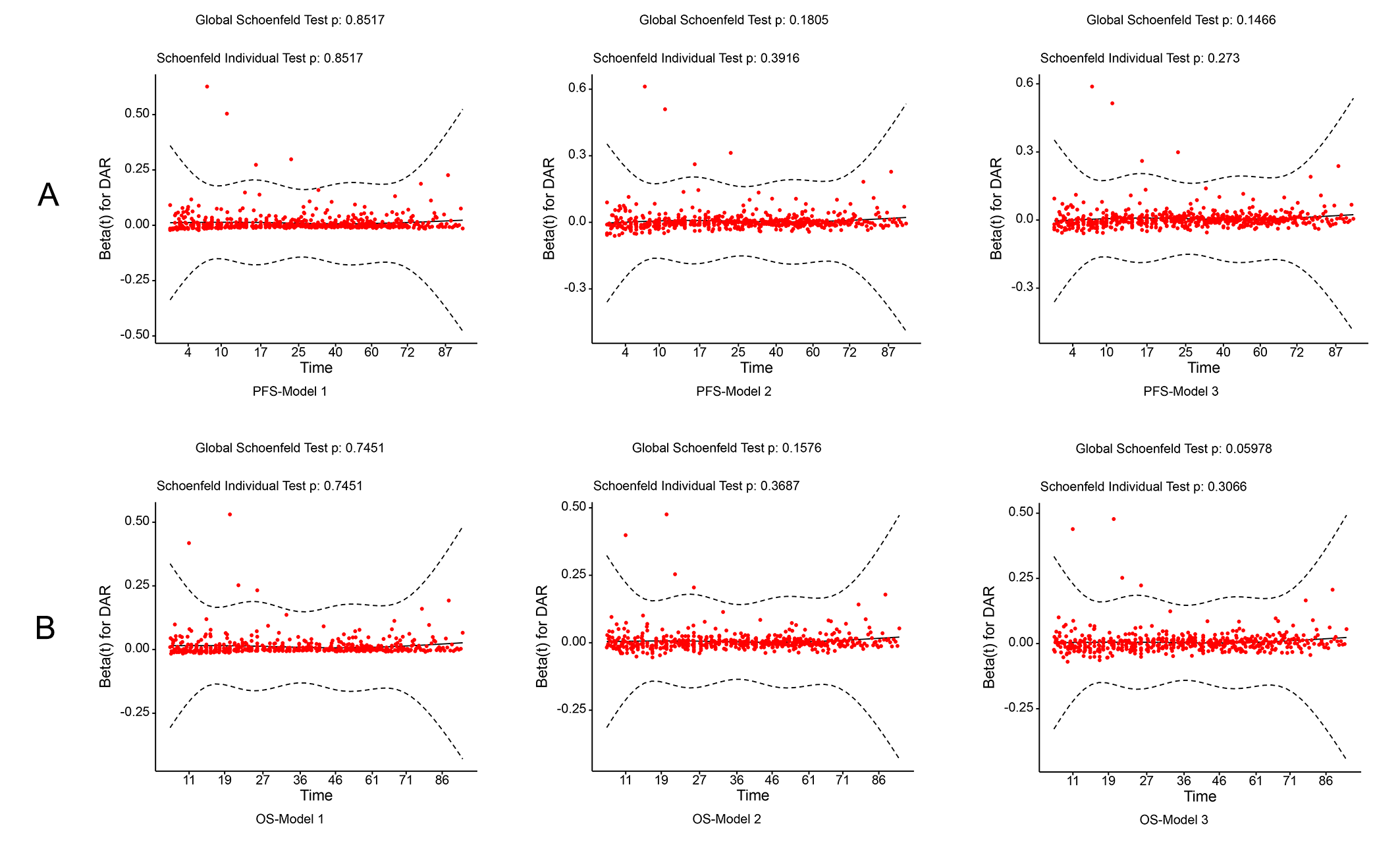
**

**Notes:** A, PFS; B, OS.

**Abbreviation:** DAR, D-Dimer to Albumin Ratio; PFS, Progression-free survival; OS, Overall survival.

**Figure S8.** The association between DAR and hazard ratio of PFS/OS in various subgroups.


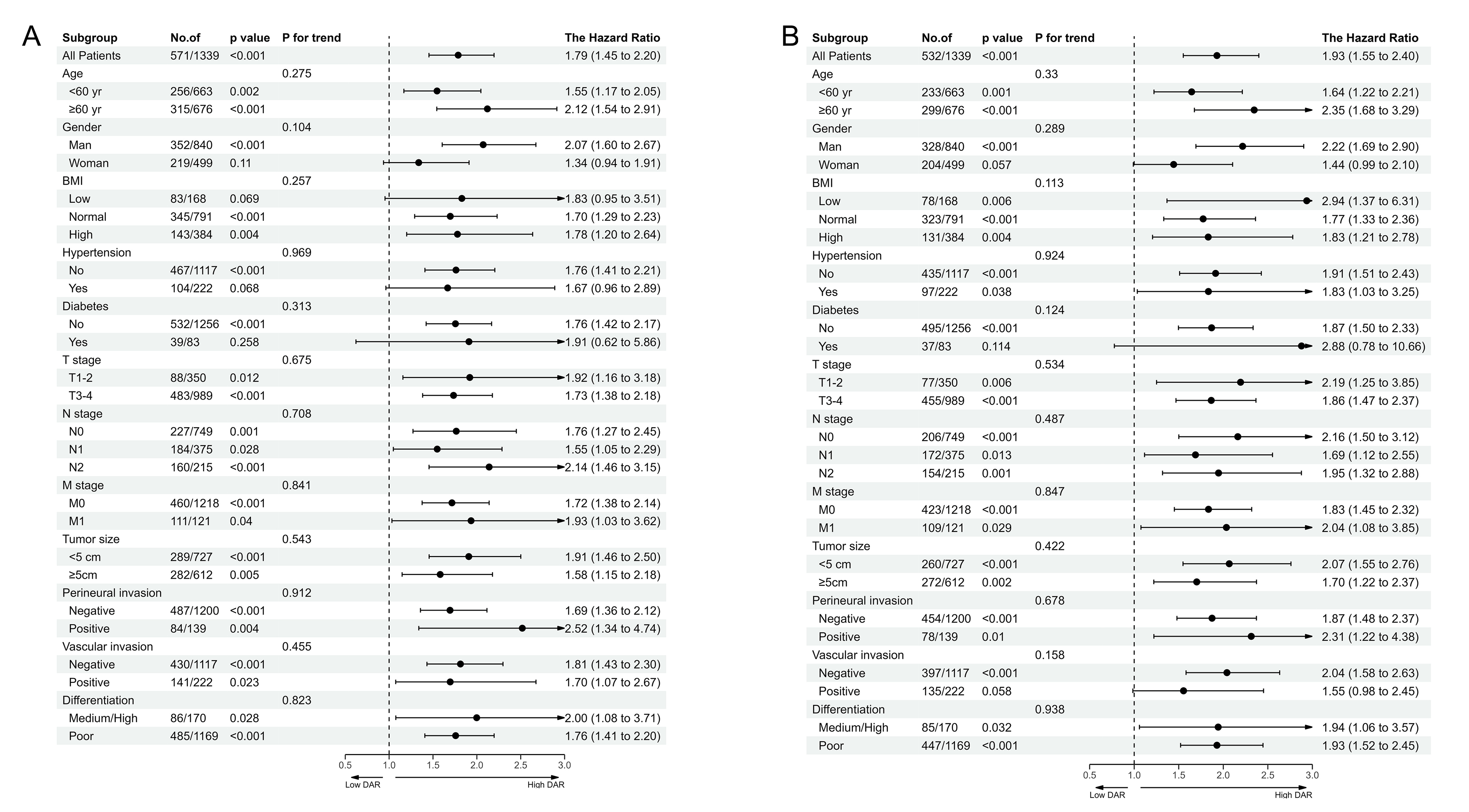


**Notes:** A, PFS; B, OS.

**Abbreviation:** DAR, D-Dimer to Albumin Ratio; PFS, Progression-free survival; OS, Overall survival; HR, Hazard ratio; CI, Confidence interval.

**Figure S9.** Construction the PFS nomogram in CRC patients.

**
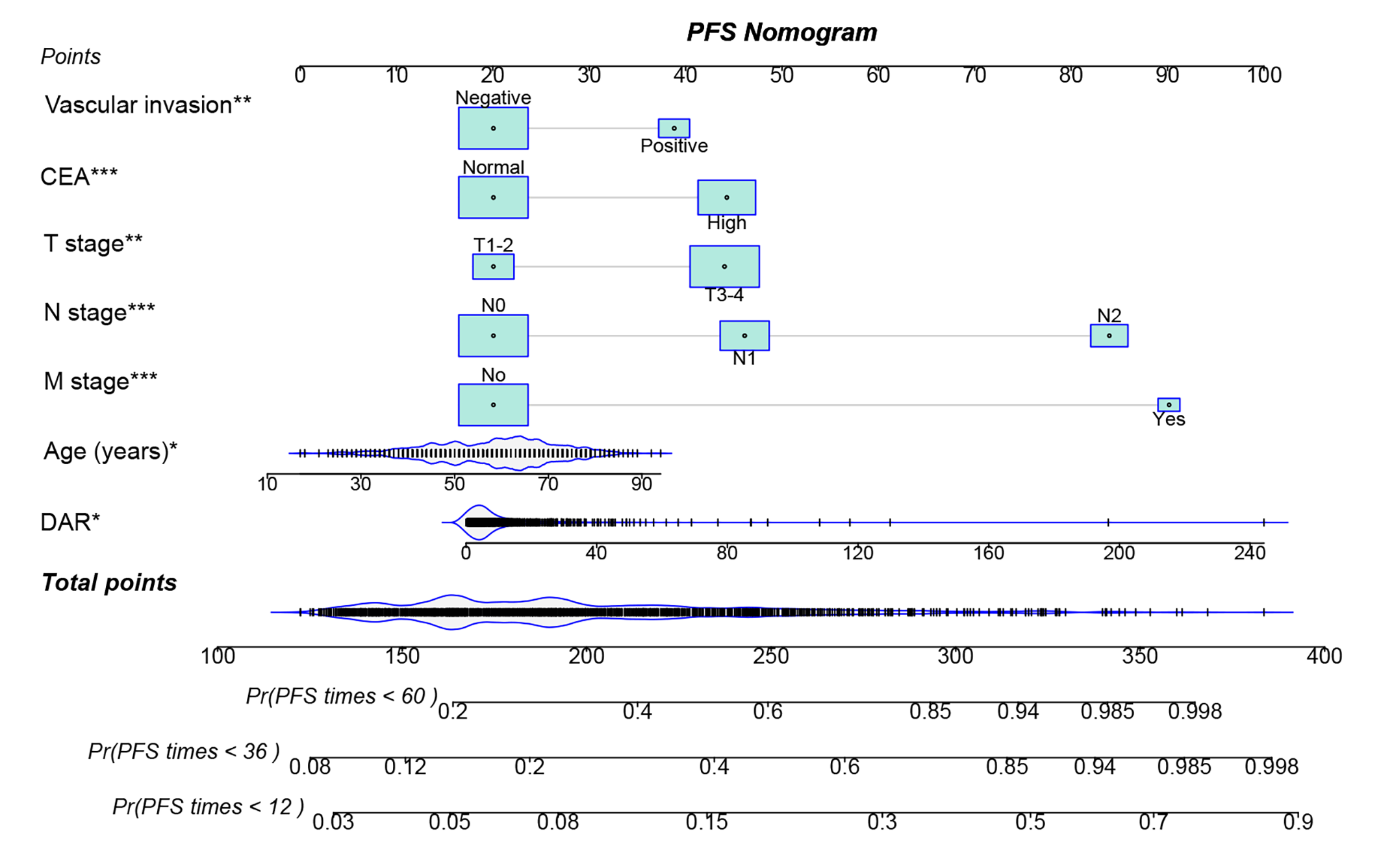
**

**Abbreviation:** DAR, D-Dimer to Albumin Ratio; PFS, Progression-free survival.

**Figure S10.** Construction the OS nomogram in CRC patients.

**
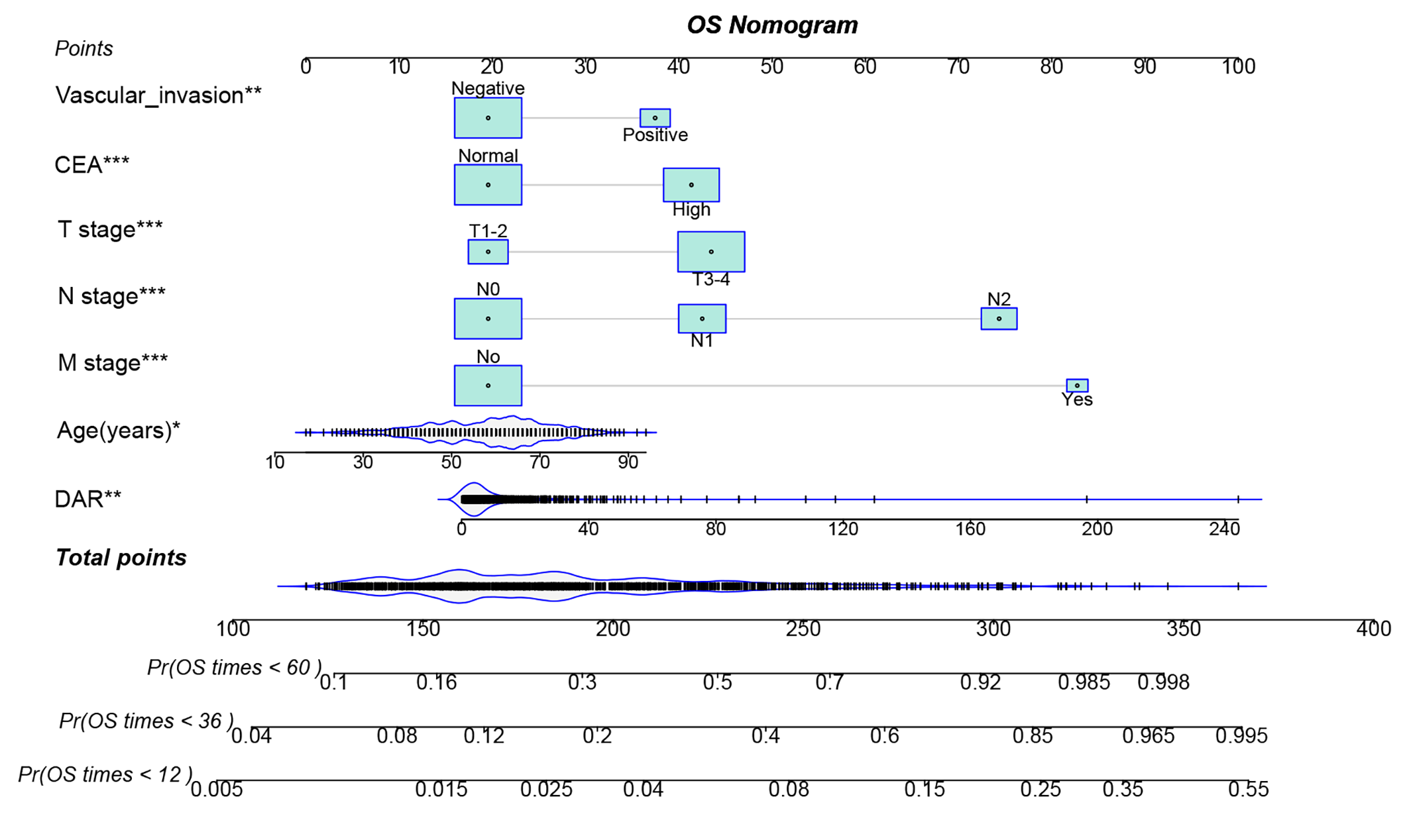
**

**Abbreviation:** DAR, D-Dimer to Albumin Ratio; OS, Overall survival.

**Figure S11.** The 1-, 3-, and 5-year ROC of PFS and OS nomograms.

**
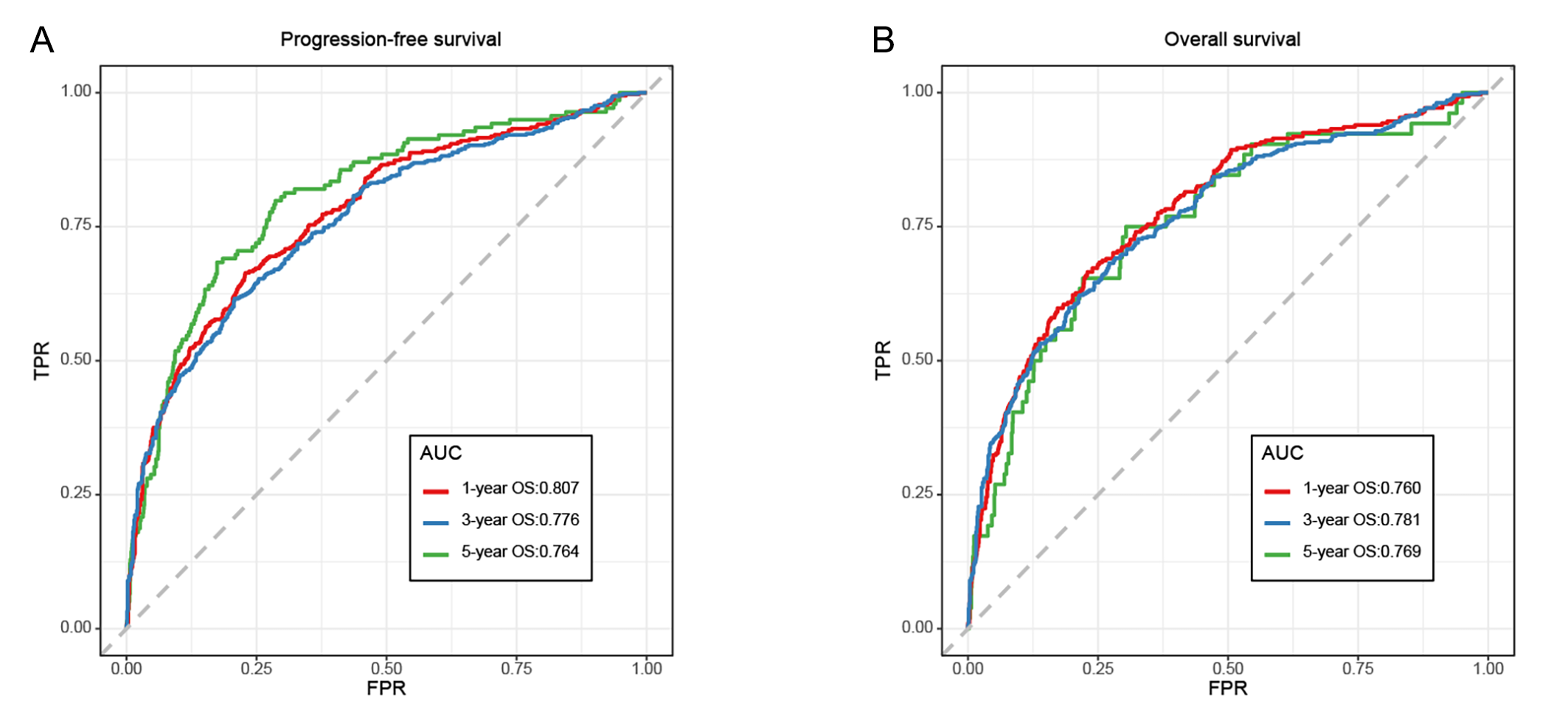
**

**Notes:** A, PFS; B, OS.

**Abbreviation:** PFS, Progression-free survival; OS, Overall survival; ROC, Receiver operator characteristic curve.

**Figure S12.** Calibration curve of the PFS/OS nomograms.

**
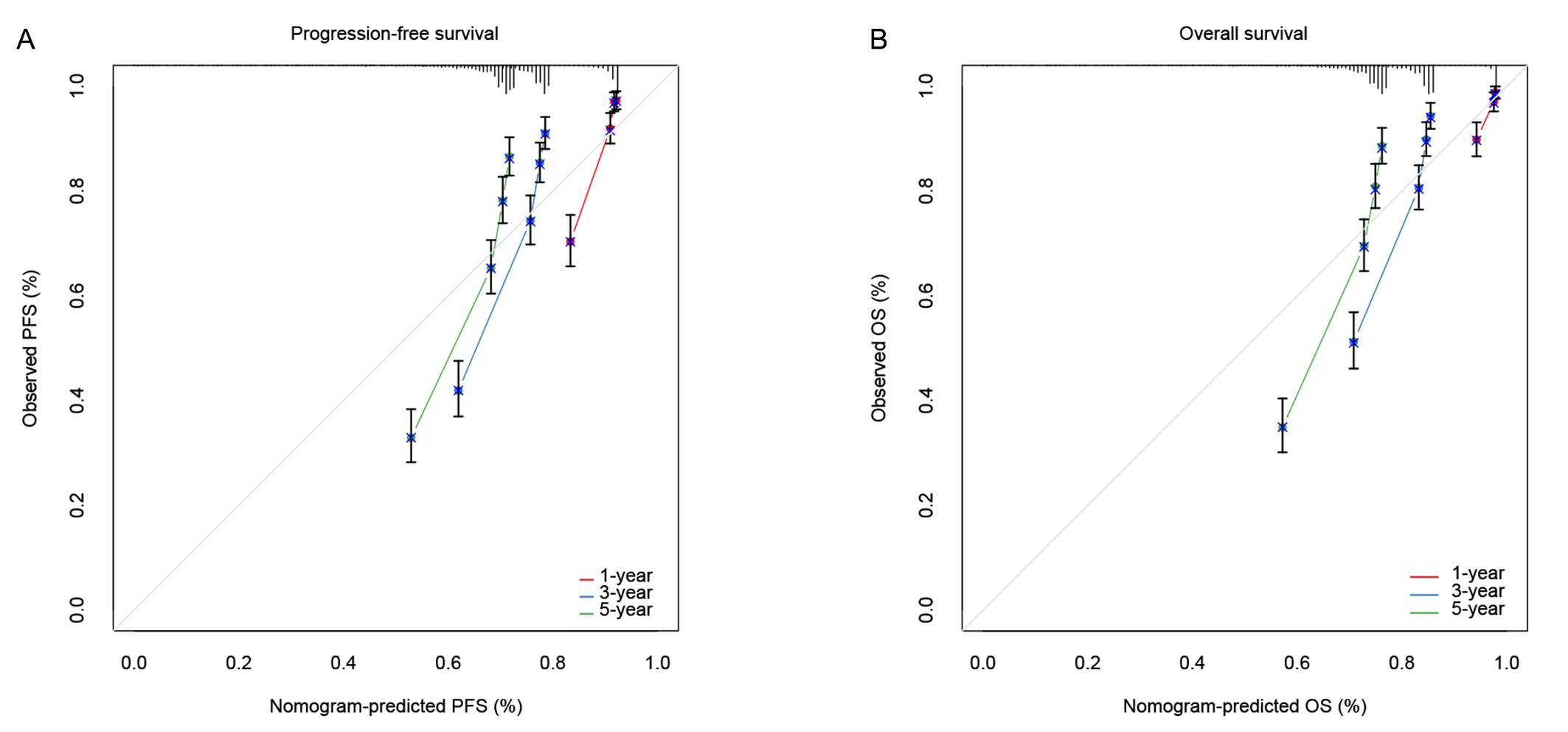
**

**Notes:** A, PFS; B, OS.

**Abbreviation:** PFS, Progression-free survival; OS, Overall survival.

**Figure S13.** The DCA of PFS/OS nomograms.

**
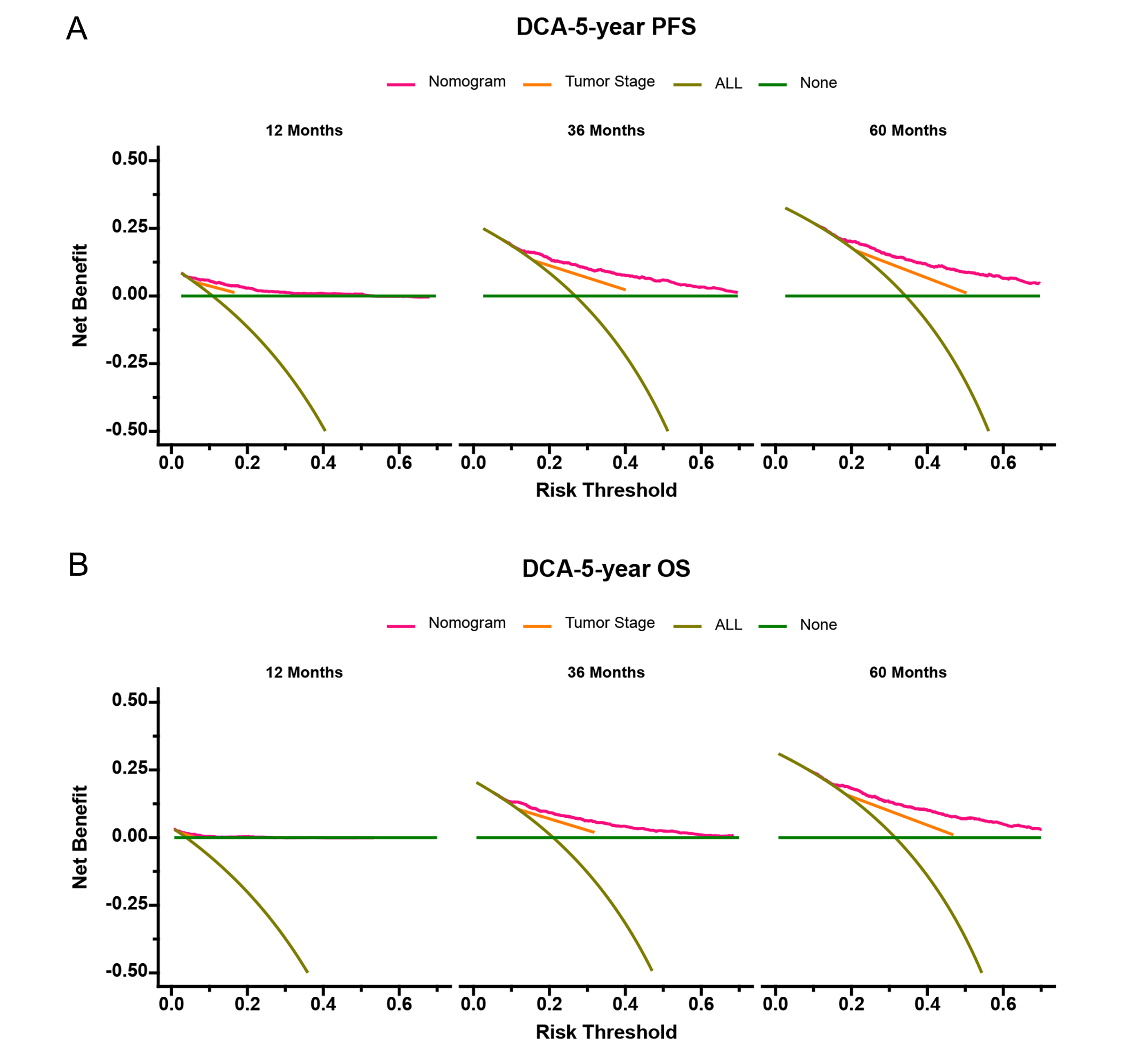
**

**Notes:** A, PFS; B, OS.

**Abbreviation:** PFS, Progression-free survival; OS, Overall survival; DCA, Decision curve analysis.

**Figure S14.** Kaplan-Meier curve of low and high score of nomograms in patients with colorectal cancer.

**
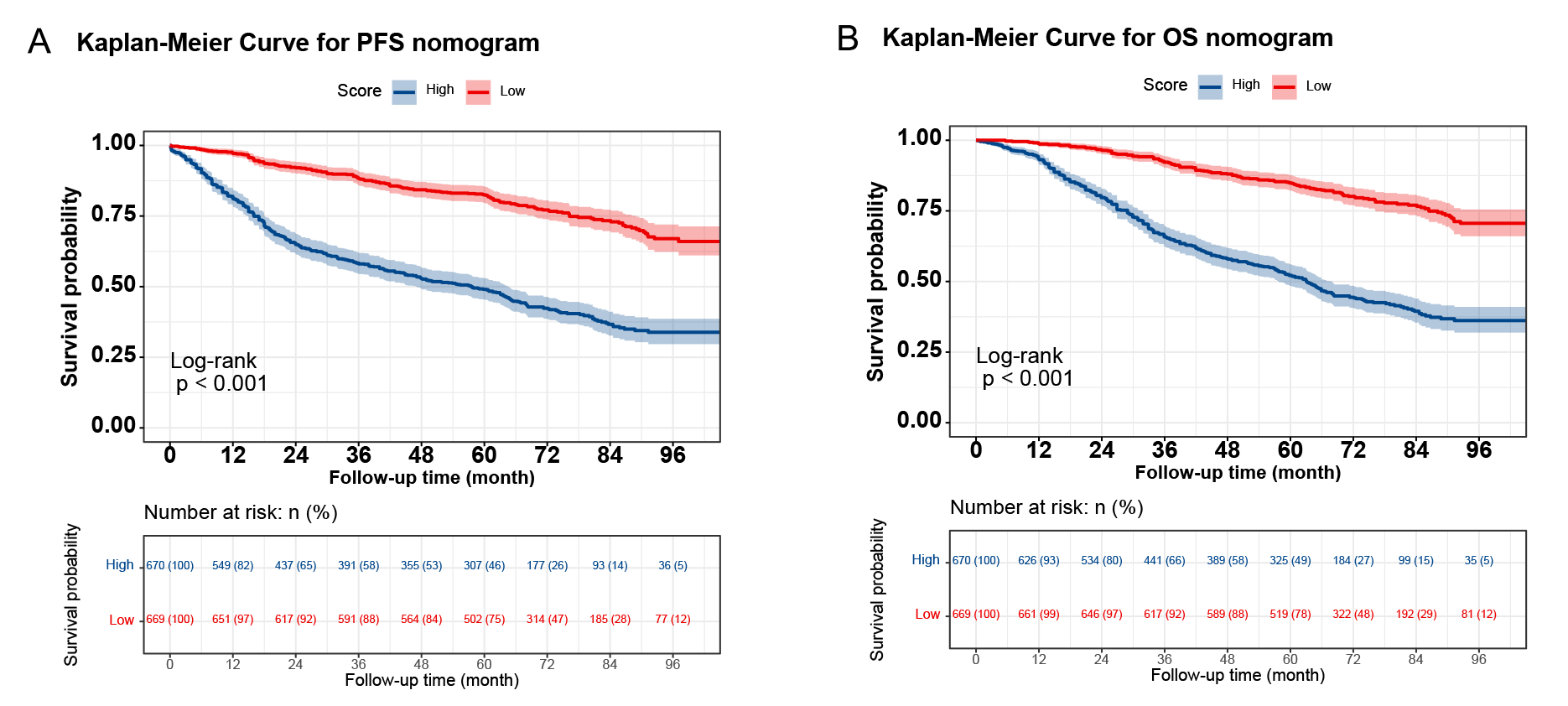
**

**Notes:** A, PFS nomogram; B, OS nomogram.

**Abbreviation:** PFS, Progression-free survival; OS, Overall survival.

**Figure S15. The ROC of Internal validation cohorts.**

**
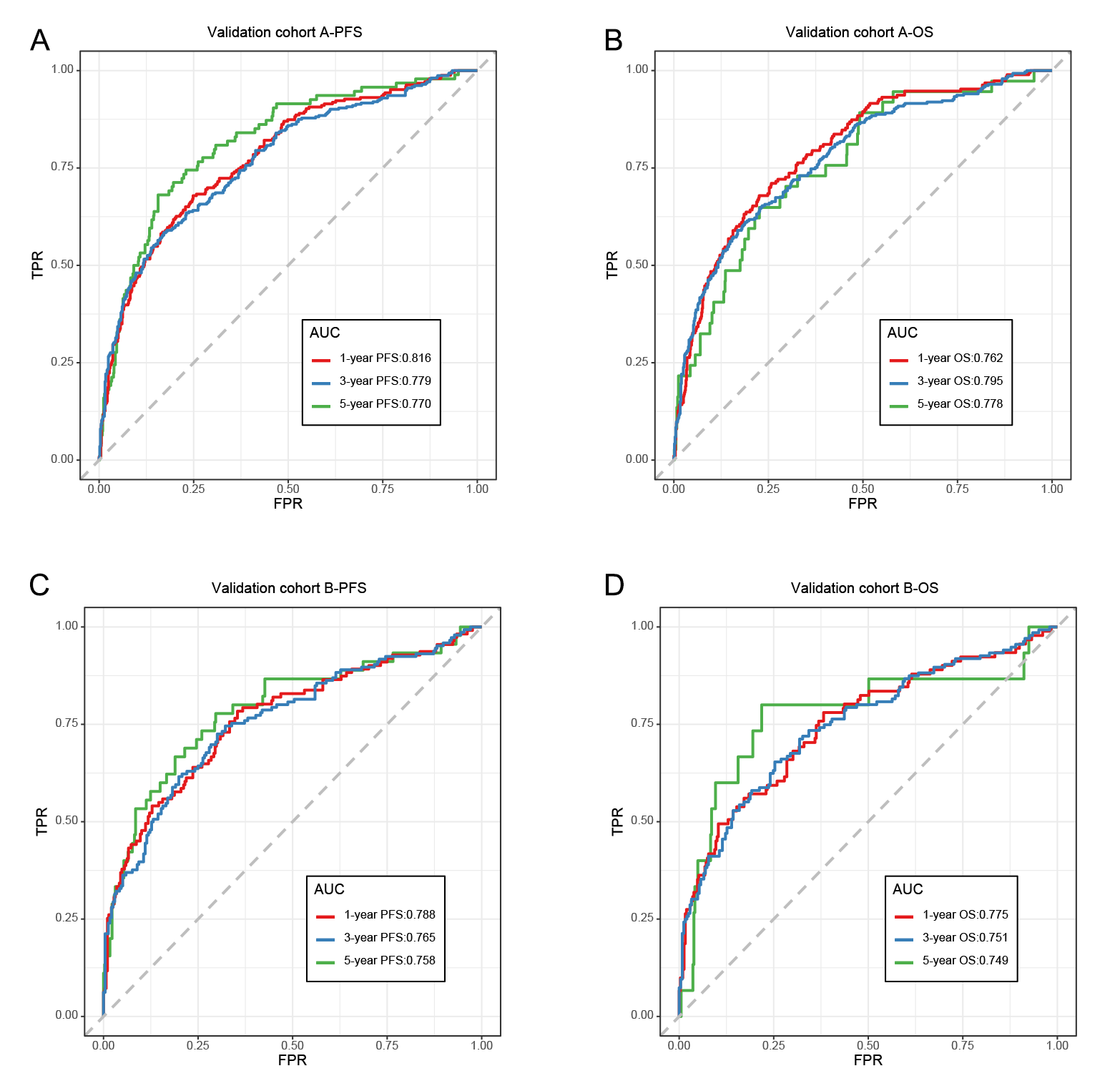
**

**Notes:** A, validation cohrt A-PFS; B, validation cohrt A-OS; C, validation cohrt B-PFS; D, validation cohrt B-OS.

**Abbreviation:** PFS, Progression-free survival; OS, Overall survival; ROC, Receiver operator characteristic curve.

**Figure S16.** Calibration curve at validation cohorts.

**
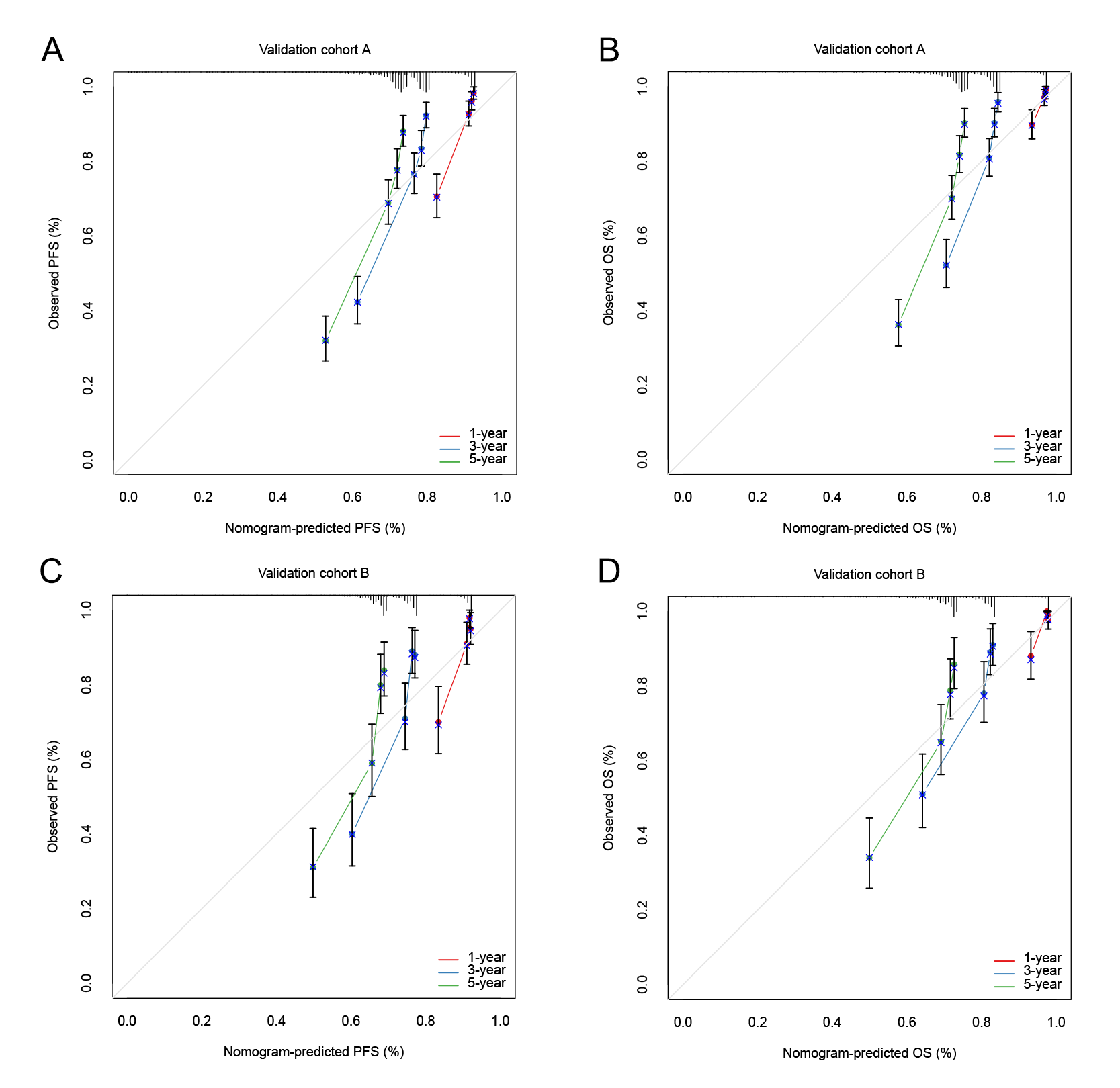
**

**Notes:** A, validation cohrt A-PFS; B, validation cohrt A-OS; C, validation cohrt B-PFS; D, validation cohrt B-OS.

**Abbreviation:** PFS, Progression-free survival; OS, Overall survival.

**Figure S17.** DCA curve at validation cohorts.

**
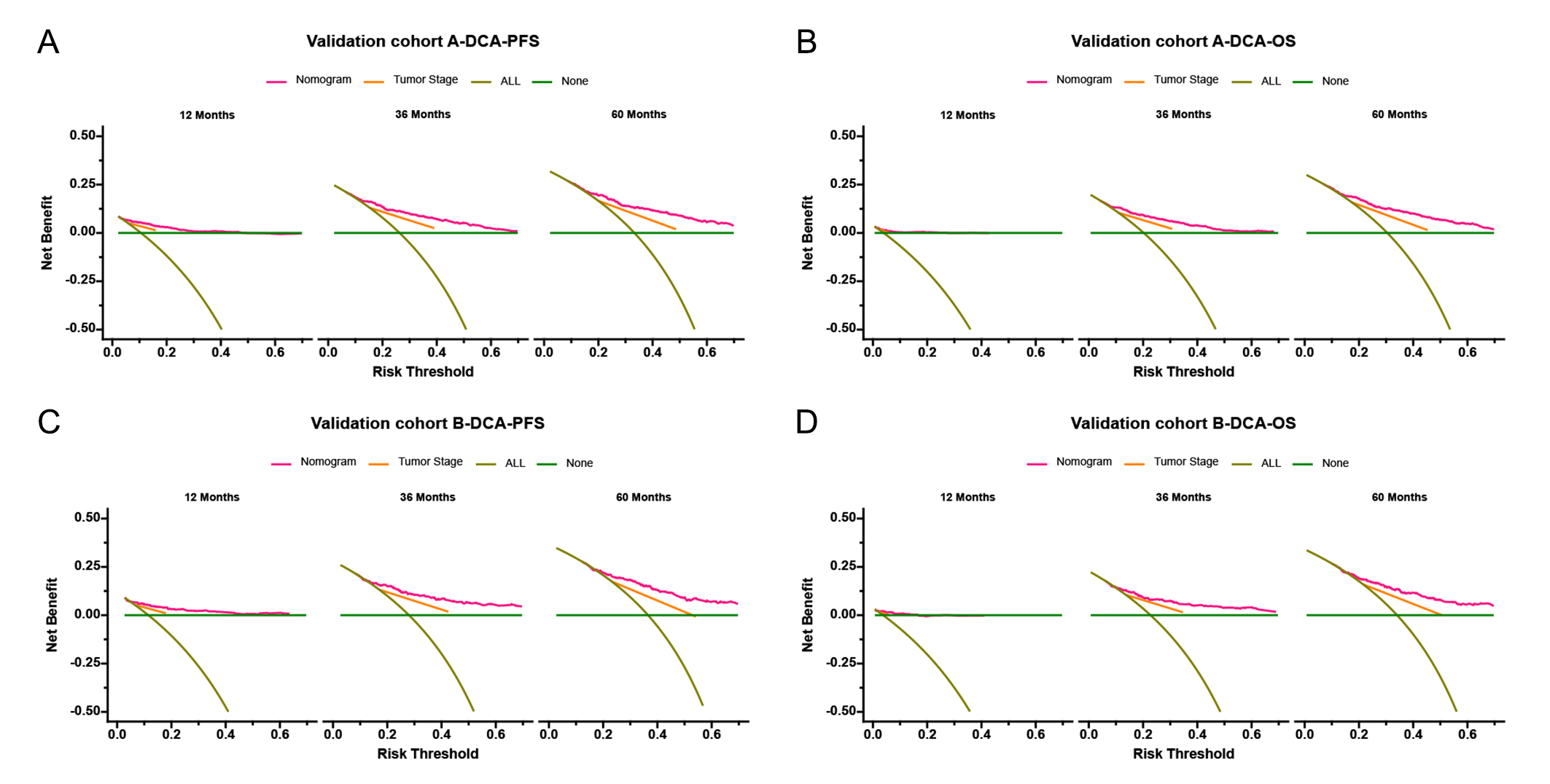
**

**Notes:** A, validation cohrt A-PFS; B, validation cohrt A-OS; C, validation cohrt B-PFS; D, validation cohrt B-OS.

**Abbreviation:** PFS, Progression-free survival; OS, Overall survival; DCA, Decision curve analysis.

**Figure S18.** Kaplan-Meier curve of low and high score of nomograms at validation cohorts.

**
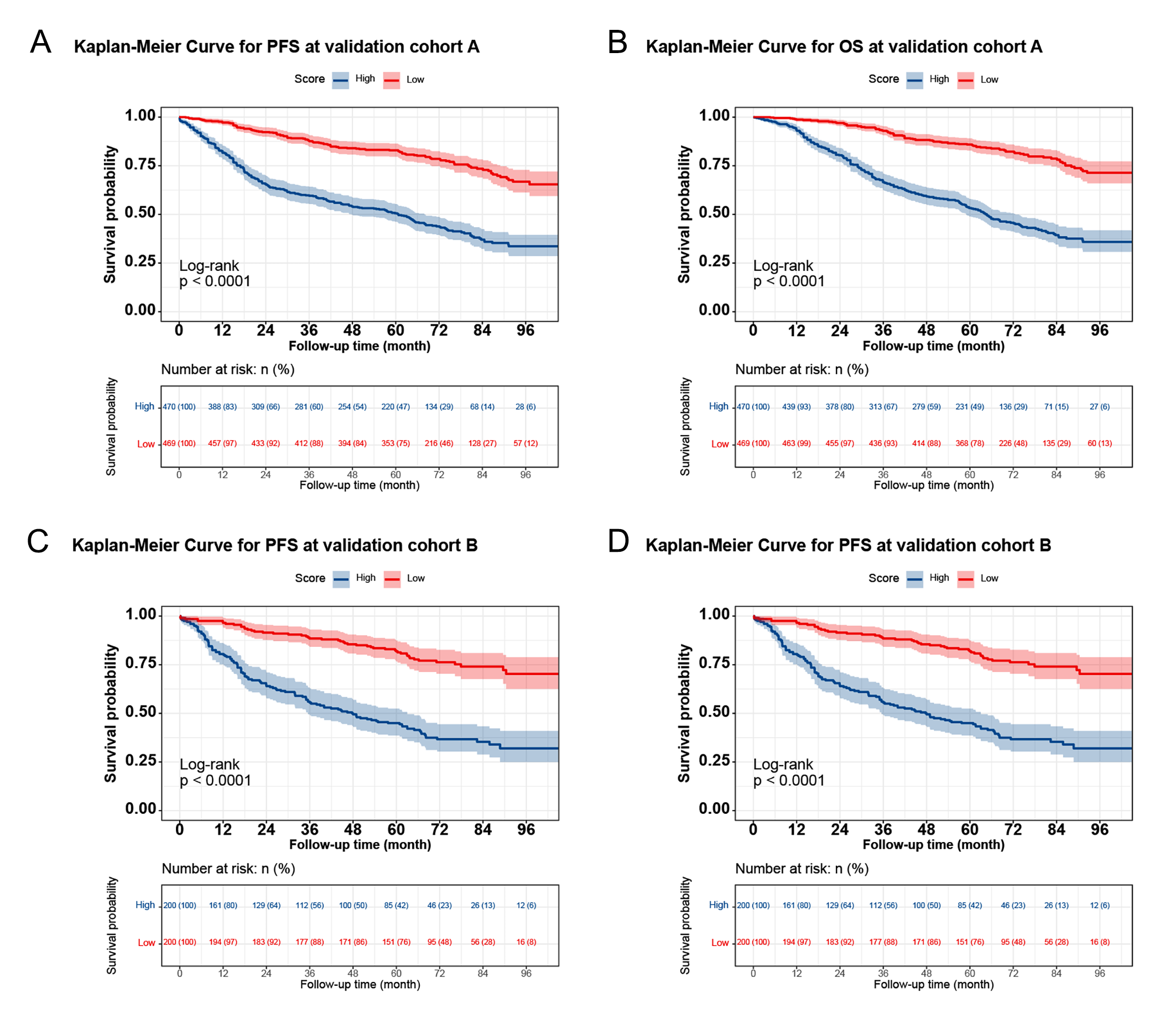
**

**Notes:** A, validation cohrt A-PFS; B, validation cohrt A-OS; C, validation cohrt B-PFS; D, validation cohrt B-OS.

**Abbreviation:** PFS, Progression-free survival; OS, Overall survival.

**Table S1** Clinicopathological characteristics of patients with colorectal cancer.

| Clinicopathological characteristics | Overall | Low DAR | High DAR | p |
| --- | --- | --- | --- | --- |
|  | n=1339 | n=470 | n=869 |  |
| Sex (Man) | 840 (62.7) | 322 (68.5) | 518 (59.6) | 0.002 |
| Age (mean (SD)) | 58.28 (12.93) | 54.49 (12.26) | 60.33 (12.83) | <0.001 |
| BMI (median [IQR]) | 22.07 (19.98, 24.44) | 22.67 (20.55, 25.15) | 21.79 (19.61, 23.98) | <0.001 |
| Hypertension (Yes) | 222 (16.6) | 69 (14.7) | 153 (17.6) | 0.195 |
| Diabetes (Yes) | 83 ( 6.2) | 24 ( 5.1) | 59 ( 6.8) | 0.271 |
| T stage (T3-4) | 989 (73.9) | 320 (68.1) | 669 (77.0) | 0.001 |
| N stage |  |  |  | 0.275 |
| N0 | 749 (55.9) | 264 (56.2) | 485 (55.8) |  |
| N1 | 375 (28.0) | 122 (26.0) | 253 (29.1) |  |
| N2 | 215 (16.1) | 84 (17.9) | 131 (15.1) |  |
| M stage | 121 ( 9.0) | 23 ( 4.9) | 98 (11.3) | <0.001 |
| TNM stage (III-IV) | 626 (46.8) | 211 (44.9) | 415 (47.8) | 0.345 |
| Perineural invasion (Yes) | 139 (10.4) | 42 ( 8.9) | 97 (11.2) | 0.238 |
| Vascular invasion (Yes) | 222 (16.6) | 73 (15.5) | 149 (17.1) | 0.496 |
| Differentiation (Poor) | 170 (12.7) | 57 (12.1) | 113 (13.0) | 0.709 |
| Location (Rectal cancer) | 706 (52.7) | 308 (65.5) | 398 (45.8) | <0.001 |
| Tumor size (median [IQR]) | 4.81 (2.09) | 4.14 (1.65) | 5.17 (2.21) | <0.001 |
| CEA (median [IQR]) | 3.83 (2.04, 10.69) | 3.04 (1.62, 6.78) | 4.29 (2.32, 14.29) | <0.001 |
| Radiotherapy (%) | 130 ( 9.7) | 75 (16.0) | 55 ( 6.3) | <0.001 |
| Chemotherapy (%) | 608 (45.4) | 230 (48.9) | 378 (43.5) | 0.064 |
| Death (Yes) | 532 (39.7) | 123 (26.2) | 409 (47.1) | <0.001 |
| Recurrence (Yes) | 368 (27.5) | 103 (21.9) | 265 (30.5) | 0.001 |
| Length of stay (median [IQR]) | 17.00 (12.00, 21.00) | 16.00 (11.00, 20.00) | 17.00 (12.00, 21.00) | 0.001 |
| Hospitalization cost (median [IQR]) | 49578.48 (44628.92, 55838.04) | 48426.69 (44081.48, 54145.13) | 50625.64 (45075.15, 57693.55) | <0.001 |

**Table Note:** CRC, colorectal cancer; DAR, D-Dimer to Albumin Ratio; BMI, body mass index.

**Table S2**. Univariate and multivariate Cox regression analysis of clinicopathological characteristics associated with progression-free survival in CRC patients.

| Characteristic | Progression-free survival | | | |
| --- | --- | --- | --- | --- |
|  | Univariate analysis | | Multivariate analysis | |
|  | HR (95%CI) | P value | HR (95%CI) | P value |
| Age | 1.294 (1.097-1.526) | 0.002 | 1.236 (1.04 - 1.468) | 0.016 |
| T stage | 2.35 (1.873-2.95) | <0.001 | 1.43 (1.122 - 1.823) | 0.004 |
| N stage |  | <0.001 |  | <0.001 |
| N0 | Ref. |  | Ref. |  |
| N1 | 1.883 (1.55-2.287) | <0.001 | 1.523 (1.245 - 1.863) | <0.001 |
| N2 | 4.116 (3.356-5.047) | <0.001 | 2.995 (2.392 - 3.749) | <0.001 |
| M stage | 5.361 (4.34-6.623) | <0.001 | 3.198 (2.561 - 3.995) | <0.001 |
| Perineural invasion (Yes) | 1.781 (1.413-2.246) | <0.001 | 1.071 (0.828 - 1.385) | 0.602 |
| Vascular invasion (Yes) | 2.095 (1.731-2.535) | <0.001 | 1.317 (1.058 - 1.639) | 0.014 |
| Differentiation (high/medium) | 0.699 (0.556-0.879) | 0.002 | 0.843 (0.666 - 1.069) | 0.159 |
| Tumor size (≥5cm) | 1.203 (1.021-1.418) | 0.027 | 0.927 (0.781 - 1.101) | 0.388 |
| CEA (≥5ng/ml) | 1.965 (1.667-2.317) | <0.001 | 1.432 (1.201 - 1.708) | <0.001 |
| DAR (High) | 1.754 (1.452-2.119) | <0.001 | 1.57 (1.286 - 1.917) | <0.001 |

**Table Note:** CRC, colorectal cancer; DAR, D-Dimer to Albumin Ratio.

**Table S3** Univariate and multivariate Cox regression analysis of clinicopathological characteristics associated with overall survival in CRC patients.

| Characteristic | Overall survival | | | |
| --- | --- | --- | --- | --- |
|  | Univariate analysis | | Multivariate analysis | |
|  | HR (95%CI) | P value | HR (95%CI) | P value |
| Age | 1.354 (1.141-1.607) | 0.001 | 1.244 (1.041 - 1.488) | 0.016 |
| T stage | 2.493 (1.958-3.175) | <0.001 | 1.464 (1.132 - 1.894) | 0.004 |
| N stage |  | <0.001 |  | <0.001 |
| N0 | Ref. |  |  |  |
| N1 | 1.903 (1.554-2.331) | <0.001 | 1.533 (1.243 - 1.89) | <0.001 |
| N2 | 4.174 (3.382-5.151) | <0.001 | 2.926 (2.319 - 3.692) | <0.001 |
| M stage | 5.526 (4.458-6.849) | <0.001 | 3.29 (2.626 - 4.122) | <0.001 |
| Perineural invasion (Yes) | 1.737 (1.366-2.21) | <0.001 | 1.013 (0.776 - 1.323) | 0.923 |
| Vascular invasion (Yes) | 2.142 (1.761-2.606) | <0.001 | 1.355 (1.082 - 1.696) | 0.008 |
| Differentiation (high/medium) | 0.644 (0.51-0.812) | <0.001 | 0.763 (0.6 - 0.97) | 0.027 |
| Tumor size (≥5cm) | 1.327 (1.12-1.573) | 0.001 | 1.017 (0.852 - 1.215) | 0.852 |
| CEA (≥5ng/ml) | 2.021 (1.704-2.396) | <0.001 | 1.424 (1.187 - 1.709) | <0.001 |
| DAR (High) | 1.987 (1.624-2.431) | <0.001 | 1.76 (1.423 - 2.176) | <0.001 |

**Table Note:** CRC, colorectal cancer; DAR, D-Dimer to Albumin Ratio.

**Table S4** Clinicopathological characteristics of validation cohorts with colorectal cancer.

| Clinicopathological characteristics | Validation cohort A | Validation cohort B | p |
| --- | --- | --- | --- |
|  | n=939 | n=400 |  |
| Sex (Man) | 599 (63.8) | 241 (60.2) | 0.244 |
| Age (mean (SD)) | 58.77 (12.58) | 57.13 (13.67) | 0.034 |
| BMI (median [IQR]) | 22.15 (19.96, 24.54) | 22.03 (20.04, 24.22) | 0.934 |
| Hypertension (Yes) | 150 (16.0) | 72 (18.0) | 0.405 |
| Diabetes (Yes) | 54 ( 5.8) | 29 ( 7.2) | 0.359 |
| T stage (T3-4) | 697 (74.2) | 292 (73.0) | 0.689 |
| N stage |  |  | 0.337 |
| N0 | 514 (54.7) | 235 (58.8) |  |
| N1 | 267 (28.4) | 108 (27.0) |  |
| N2 | 158 (16.8) | 57 (14.2) |  |
| M stage | 87 ( 9.3) | 34 ( 8.5) | 0.732 |
| TNM stage (III-IV) | 450 (47.9) | 176 (44.0) | 0.209 |
| Perineural invasion (Yes) | 105 (11.2) | 34 ( 8.5) | 0.169 |
| Vascular invasion (Yes) | 154 (16.4) | 68 (17.0) | 0.85 |
| Differentiation (Poor) | 107 (11.4) | 63 (15.8) | 0.036 |
| Location (Rectal cancer) | 491 (52.3) | 215 (53.8) | 0.667 |
| Tumor size (median [IQR]) | 4.81 (2.08) | 4.81 (2.12) | 0.971 |
| CEA (median [IQR]) | 3.94 (2.10, 10.89) | 3.47 (1.87, 9.83) | 0.191 |
| Radiotherapy (%) | 89 ( 9.5) | 41 (10.2) | 0.737 |
| Chemotherapy (%) | 421 (44.8) | 187 (46.8) | 0.559 |
| Death (Yes) | 365 (38.9) | 167 (41.8) | 0.355 |
| Recurrence (Yes) | 252 (26.8) | 116 (29.0) | 0.457 |
| Length of stay (median [IQR]) | 17.00 (11.00, 21.00) | 17.00 (12.00, 21.00) | 0.65 |
| Hospitalization cost (median [IQR]) | 49213.67 (44561.49, 55376.72) | 50900.27 (45049.44, 58156.30) | 0.031 |

**Table Note:** CRC, colorectal cancer; DAR, D-Dimer to Albumin Ratio; BMI, body mass index.
